# Evaluation of New Dihydrophthalazine-Appended 2,4-Diaminopyrimidines against *Bacillus anthracis*: Improved Syntheses Using a New Pincer Complex

**DOI:** 10.3390/molecules20047222

**Published:** 2015-04-21

**Authors:** Nagendra Prasad Muddala, Baskar Nammalwar, Subhashini Selvaraju, Christina R. Bourne, Mary Henry, Richard A. Bunce, K. Darrell Berlin, Esther W. Barrow, William W. Barrow

**Affiliations:** 1 Department of Chemistry, Oklahoma State University, 107 Physical Sciences, Stillwater, OK 74078, USA; E-Mails: muddala@okstate.edu (N.P.M.); baskar@okstate.edu (B.N.); subhas@okstate.edu (S.S.); kdb@okstate.edu (K.D.B.); 2 Department of Chemistry and Biochemistry, University of Oklahoma, 101 Stephenson Parkway, Norman, OK 73019, USA; E-Mail: cbourne@ou.edu; 3 Department of Veterinary Pathobiology, Oklahoma State University, 250 McElroy Hall, Stillwater, OK 74078, USA; E-Mails: henrym@okstate.edu (M.H.); esther.barrow@okstate.edu (E.W.B.); bill.barrow@okstate.edu (W.W.B.)

**Keywords:** 2,4-diaminopyrimidine antifolates, dihydrofolate reductase (DHFR), *Bacillus anthracis*, pincer complexes, Heck coupling

## Abstract

The synthesis and evaluation of ten new dihydrophthalazine-appended 2,4-diaminopyrimidines as potential drugs to treat *Bacillus anthracis* is reported. An improved synthesis utilizing a new pincer catalyst, dichlorobis[1-(dicyclohexylphosphanyl)-piperidine]palladium(II), allows the final Heck coupling to be performed at 90 °C using triethylamine as the base. These milder conditions have been used to achieve improved yields for new and previously reported substrates with functional groups that degrade or react at the normal 140 °C reaction temperature. An analytical protocol for separating the *S* and *R* enantiomers of two of the most active compounds is also disclosed. Finally, the X-ray structure for the most active enantiomer of the lead compound, (*S*)-RAB1, is given.

## 1. Introduction

*Bacillus anthracis*, a Gram-positive, non-motile bacterium distributed as spores, is the etiologic agent responsible for deadly anthrax in both humans and animals [1,2]. The Center for Disease Control and Prevention (CDC) lists *B. anthracis* spores as a category A agent which can spread to individuals via gastrointestinal, cutaneous, injection, or inhalation routes and is considered a serious bioweapon threat [3,4,5]. Due to the worldwide emergence of antibiotic resistance, these pathogens can be engineered against current available drugs for mass destruction purposes [6,7,8,9]. Thus, there is an imminent need to develop new antibiotics to counteract this organism in case of a bioterror event [10].

In the last 50 years, inhibition of dihydrofolate reductase enzyme (DHFR) in the folate pathway has been a focus for the development of various antibacterial drugs [11,12]. With the advent of the drug trimethoprim (TMP), which demonstrated higher inhibition (5 log10) of bacterial DHFR compared to mammalian DHFR, many researchers targeted these enzymes to develop medicinal agents with better pharmacokinetics [11].

**Figure 1:**
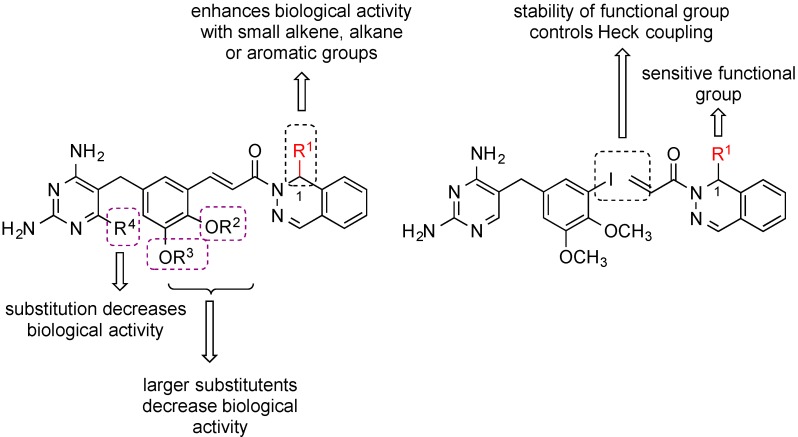
Modification of DAP inhibitors.

Some bacteria have proven resistant to TMP due to encoded differences in the chromosomal DHFR sequence, which makes it an ineffective drug. *B. anthracis* is one such bacterium, which presumably has innate resistance to TMP due to the poor binding affinity of the drug with DHFR. Due to the increased concern regarding bioterrorism and engineered drug-resistant *B. anthracis* strains, a new method to treat this bacterium has been sought. Over the last five years, our research group has developed substituted dihydrophthalazine-appended 2,4-diaminopyrimidines (DAP inhibitors) as modified TMP derivatives for effective inhibition of this organism [13,14,15]. These structures have shown inhibition of *B.* *anthracis* at 0.5–2 μg/mL concentrations. In pursuit of identifying a drug with enhanced activity and bioavailability, we previously developed first and second generation DAP inhibitors. In the current project, a family of compounds with changes at the R^1^ position of the ring system has been prepared and studied (see Figure 1). It was observed earlier that any alteration of R^1^, at the C-1 stereocenter of the dihydrophthalazine unit, tended to modulate interaction of the protein surface with the surrounding solvent, and thus, the inhibitory activity [13,15]. Alternatively, changes at the R^2^, R^3^ and R^4^ positions on the ring disrupted the compound orientation in the binding pocket, which resulted in attenuated potency [14,16]. Numerous compounds with changes at the R^1^ position were synthesized and evaluated, but derivatives with sensitive functional groups at this position proved challenging to prepare. Such groups often underwent decomposition at high temperatures leading to an increased impurity profile and decreased yields of the targeted products. Hence, isolation and purification remained tedious with respect to these substrates. To overcome this difficulty, a highly active, reliable, versatile, functional group tolerant catalyst, which could perform the final Heck coupling reaction at lower temperature was sought.

**Figure 2:**
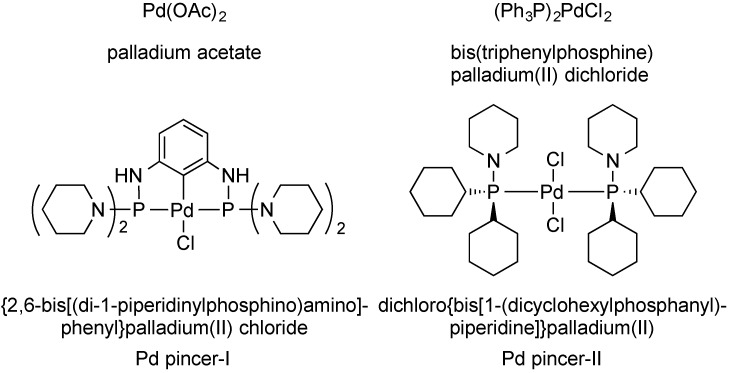
Pd catalysts for Heck coupling.

Over the past decade, a number of palladium pincer complexes with high temperature and moisture tolerance have been used in various Heck couplings [17,18,19]. These pincer complexes have played an important role in cross-coupling aryl/alkyl halides with alkenes to create new C-C bonds [20]. The recent development of pincer complexes has permitted milder reaction conditions, better substrate scope, and lower catalyst loading to afford cleaner products in higher yields. Frech and coworkers have synthesized dichlorobis[1-(dicyclohexylphosphanyl)piperidine]palladium(II) (Pd pincer-II, see Figure 2), which performs arylations of olefins using a wide variety of substrates, including activated, unactivated and deactivated systems, as well as heterocyclic aryl bromides and various substituted olefins [21,22]. This aminophosphine palladium complex offers a superior conversion rate by forming palladium nanoparticles and functions as a homogeneous catalyst. Hence, we chose the Pd pincer-II over the Pd pincer-I [18,23,24] catalyst to carry out Heck couplings at lower temperatures compared with conventional palladium catalysts, which generally required 120–140 °C.

## 2. Results and Discussion

### 2.1. Chemistry

To address the issue of functional group tolerance at R^1^, the newly developed pincer catalyst (Pd pincer-II) [21,22] was evaluated along with the previously reported commercial Pd pincer-I catalyst [18,23,24], Pd(OAc)_2_, and (PPh_3_)_2_PdCl_2_. Experiments involving the coupling of **1** with **2a** to give **3a** using various catalysts, bases, and solvents at different temperatures are summarized in Table 1.

**Table 1:** Reaction optimization. 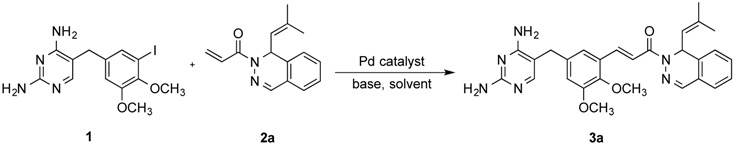

Catalyst (mol %)	Solvent	Base ^a^	Temp (°C)	Time (h)	Yield ^b^ (%)
Pd(OAc)_2_ (5.0)	DMF	NEP	90	24	35
(PPh_3_)_2_PdCl_2_ (5.0)	DMF	NEP	90	24	22
(PPh_3_)_2_PdCl_2_ (5.0)	DMF	TEA	90	24	20
Pd pincer-I (0.06)	DMF	NEP	140	18	81
Pd pincer-II (0.054)	DMF	NEP	140	12	85
Pd pincer-I (0.06)	DMF	TEA	90	18	67
**Pd pincer-II (0.054)**	**DMF**	**TEA**	**90**	**8**	**88** ^c^
Pd pincer-I (0.06)	DMF	K_2_CO_3_	90	24	NR
Pd pincer-II (0.054)	DMF	K_2_CO_3_	90	24	NR
Pd pincer-I (0.06)	DMF	NaOAc	90	24	6
Pd pincer-II (0.054)	DMF	NaOAc	90	24	NR
Pd pincer-I (0.06)	DMSO	TEA	120	18	72
Pd pincer-II (0.054)	DMSO	TEA	120	18	74
Pd pincer-I (0.06)	dioxane	TEA	110	24	NR
Pd pincer-II (0.054)	dioxane	TEA	110	24	NR
Pd pincer-I (0.06)	MeCN	NEP	90	24	NR
Pd pincer-II (0.054)	MeCN	NEP	90	24	NR
Pd pincer-I (0.06)	NMP	NEP	130	24	12
Pd pincer-II (0.054)	NMP	NEP	130	24	20
Pd pincer-I (0.06)	THF	NEP	140	24	NR
Pd pincer-II (0.054)	THF	NEP	140	24	NR

^a^ NMP = *N*-methylpyrrolidinone; NEP = *N*-ethylpiperidine; TEA = triethylamine; ^b^ NR = no reaction; ^c^ Optimized conditions.

The results revealed that 0.054 mol % of the Pd pincer-II complex gave high yields of the coupled product using TEA as the base in DMF at 90 °C. By comparison, other promoters required greater loading (up to 5 mol %), a stronger base (NEP) and higher temperature (140 °C) [18,25]. Although the Pd pincer-I complex catalyzed the coupling at a loading of 0.06 mol % using TEA at 90 °C, the reaction did not undergo complete conversion, even after extended reaction times. For all catalysts, the choice of base and solvent proved crucial. For example, the reaction did not proceed with bases such K_2_CO_3_ or NaOAc, or with solvents such as dioxane, THF, MeCN, or NMP. Moderate conversions were obtained in DMF and DMSO using NEP, but cross coupling of iodide **1** with the substituted alkene at the R^1^ position competed with the desired coupling process.

**Scheme 1:**
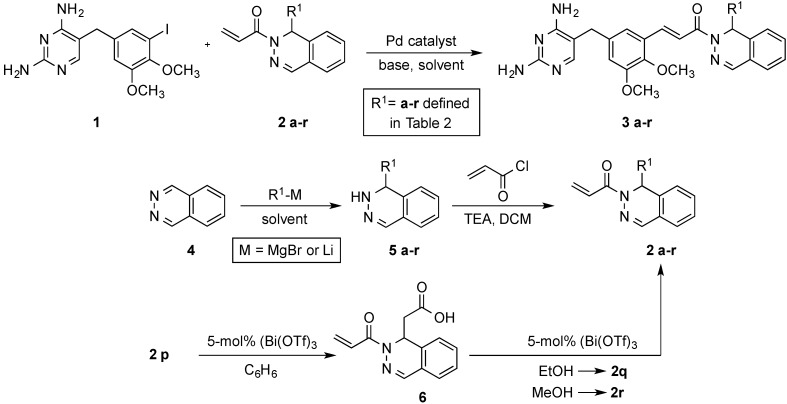
Synthesis of DAP inhibitors.

The synthesis of our DAP inhibitors **3a**–**r** is outlined in Scheme 1. The overall structures are comprised of three rings: namely, a 2,4-diaminopyrimidine, linked by a one-carbon bridge to a central dimethoxyaromatic ring, which in turn, is tethered via an acrylamide chain to N-2 of a C-1-substituted dihydrophthalazine. Precursor **1**, consisting of a 2,4-diaminopyrimidine and a dimethoxybenzyl ring, was synthesized by a two-step sequence using a previously reported strategy [15]. The substituted dihydrophthalazines **2** were obtained by addition of organolithium/magnesium reagents to phthalazine (**4**) in THF to generate racemic adducts **5a**–**p**, which were *N*-acylated with acryloyl chloride and TEA to furnish **2a**–**p** [26]. Precursors **2q** and **2r** were generated via a two-step process involving Bi(OTf)_3_ promoted hydrolysis of *tert-*butyl ester **2p** in benzene to give acid **6**, followed by re-esterification with ethyl and methyl alcohol, respectively, using catalytic Bi(OTf)_3_ [13]. Final Heck coupling of **1** and **2a**–**r** using Pd pincer-II then delivered the desired targets **3a**–**r** (Table 2).

After the optimized conditions were identified, the method was employed to install a broad range of groups at R^1^ to validate the reaction scope. A selection of alkenyl- (**3a**–**c**), alkyl- (**3d**–**e**), cycloalkyl- (**3f**–**h**), heteroaryl- (**3j**–**o**) and ester- (**3p**–**r**) substituted derivatives was prepared (see Table 2). For all cases, the reactants underwent coupling at lower temperature (90 °C) with milder base (TEA) and in shorter times (8–12 h) compared to the standard conditions (140 °C, NEP, 16 h). Compounds **3a**, **d**, **f**, **j**, **k**, **p**, **q**, **r** were previously obtained in lower yields under the standard protocol, which made the purification more tedious.

**Table 2:** Product yields.

Cpd	R^1^	Time (h) (h)	Yield (%)
**3a**	CH=C(CH_3_)_2_	8	88
**3b**	CH_2_-CH=CH_2_	8	81
**3c**	CH=CH_2_	8	85
**3d** ^a^	CH_2_CH_2_CH_3_	8	86
**3e**	*n*-C_5_H_11_	12	90
**3f**	*c*-C_3_H_5_	10	84
**3g**	*c*-C_4_H_7_	10	87
**3h**	*c*-C_5_H_9_	10	92
**3i**	C(CH_3_)_3_	12	83
**3j**	furan-2-yl	8	90
**3k**	thiophen-2-yl	8	81
**3l**	1-methylindol-2-yl	10	75
**3m**	benzofuran-2-yl	8	80
**3n**	benzothiophen-2-yl	10	78
**3o**	benzothiazol-2-yl	10	72
**3p**	CH_2_CO_2_C(CH_3_)_3_	8	83
**3q**	CH_2_CO_2_CH_2_CH_3_	8	80
**3r**	CH_2_CO_2_CH_3_	8	82

^a^ This compound has been previously designated as RAB1 [6].

### 2.2. Enantiomer Resolutions for (±)-**3a** and (±)-**3d** and X-ray Studies of (S)-**3d**

The addition of R^1^ at C-1 of the dihydrophthalazine ring creates a stereocenter, and thus, generates a racemic mixture of the target molecules. Co-crystallization of racemic **3a** and **3d** with the *B. anthracis* DHFR exhibited a preference for binding the (*S*)-enantiomers, which was revealed by X-ray studies; the (*R*)-isomers were not observed [6,27]. Previously, Chiral Technologies (West Chester, PA, USA) was engaged to resolve the enantiomers of **3a** and **3d**. The separation of 50 mg of material was accomplished under supercritical conditions with a 4.6 mm × 100 mm Chiralpak^®^ ADH column (5 µm particle size) using 40% EtOH/0.2% Et_2_NH/60% CO_2_ as the mobile phase at 35 °C [28]. For the current study, a new in-house method was developed to resolve these enantiomers under non-supercritical conditions using a 10 mm × 250 mm Chiralpak^®^ IA preparative HPLC column (5 µm). This column has greater stability than the Chiralpak^®^ ADH column, tolerates more solvents, and permits separation of a wider range of compounds. In the current work, the Chiralpak^®^ IA column, eluted with MeCN/MeOH/Et_2_NH (50:50:0.1) at 23 °C, permitted the resolution of 20 mg of the target molecule per run with 99% purity. The separation yielded the (*S*)- and (*R*)-isomers, in sufficient quantities for evaluation against bacterial targets and also for generating crystals suitable for X-ray diffraction studies.

**Figure 3:**
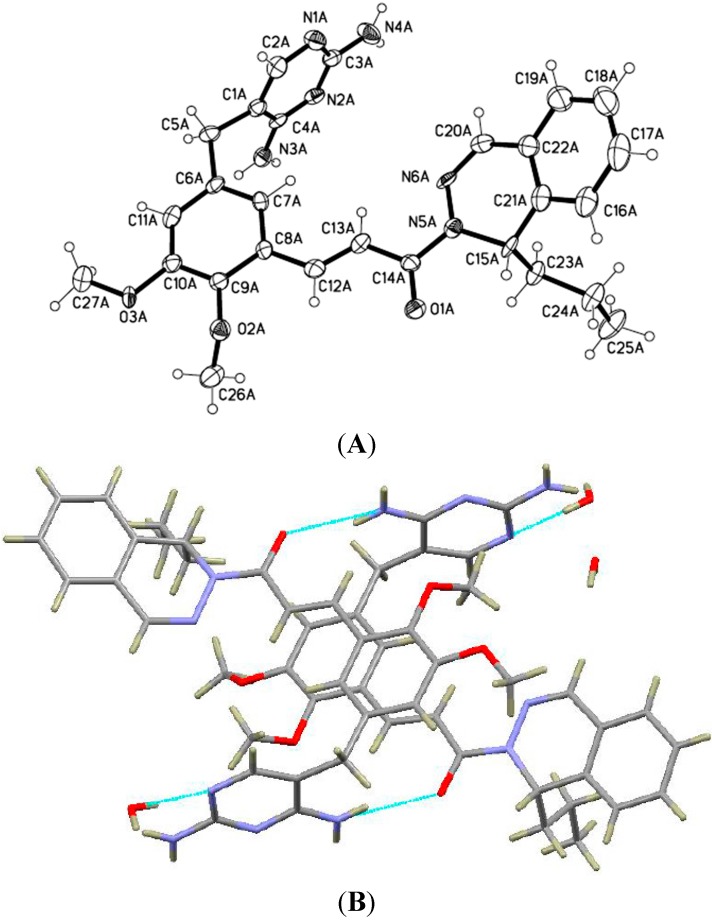
X-ray structure of (*S*)-**3d** [(*S*)-RAB1]: (**A**) Single molecule; (**B**) Unit cell

The X-ray structure of (*S*)-**3d**, previously designated as (*S*)-RAB1, is shown in Figure 3. Figure 3A shows a single molecule of the compound and Figure 3B shows the arrangement of molecules within the unit cell. The single molecule shows an angular structure, which minimizes steric interactions between functional groups on the three rings. In the unit cell, the dihydrophthalazine and dimethoxyaromatic subunits from two molecules align head-to-tail to maximize two π-stacking interactions between the electron-poor acrylamide in one molecule and the electron-rich dimethoxyaromatic ring of the other. The 2,4-diaminopyrimidine rings position themselves above and below the cage created by this stacking interaction. Additionally, the two proximal structures align to take advantage of intermolecular H-bonding interactions between the acrylamide carbonyl of one molecule and the C-4 amino group of the 2,4-diaminopyrimidine of the other. Finally, as elemental analysis has suggested, there are 2.5 water molecules in the unit cell and two of these are observed to be associated with the highly polar diaminopyrimidine rings.

### 2.3. Biology

The biological activities for the new subset of DAP-based inhibitors are summarized in Table 3. These activities were evaluated employing whole cell bacterial cultures using the standard Clinical Laboratory Standards Institute guidelines. The values are reported in minimum inhibitory concentrations (MIC) in μg/mL [29]. The enzymatic activities were evaluated using purified *B. anthracis* DHFR protein in a standardized assay yielding the concentration required to inhibit the half maximal enzyme activity rates. This IC_50_ value (data not given) was used in combination with the substrate affinity of the DHFR enzyme, in this case the K_M_ for dihydrofolate, to derive the inhibition constants (K_i_) reported in Table 3.

**Table 3:** MIC values of the inhibitors against *B. anthracis* and K_i_ values for *B. anthracis* DHFR.

Cpd	R^1^	MIC (μ/mL) *B. anthracis*	K_i_ (nM ± SEM) *B. anthracis* DHFR
(*R*)-3a *	(*R*)-CH=C(CH_3_)_2_	8	2631
(*S*)-3a *	(*S*)-CH=C(CH_3_)_2_	0.5	4.4 ± 0.2
(*RS*)-**3b**	CH_2_-CH=CH_2_	4	8.2 ± 0.3
(*RS*)-**3c**	CH=CH_2_	1–2	3.2 ± 0.2
(*R*)-3d *	(*R*)-CH_2_CHCH_3_	8	>2870
(*S*)-3d *	(*S*)-CH_2_CHCH_3_	0.25–0.5	5.0 ± 0.5
(*RS*)-**3e**	*n*-C_5_H_11_	4	5.5 ± 0.2
(*RS*)-**3g**	*c*-C_4_H_7_	4	5.5 ± 0.2
(*RS*)-**3h**	*c*-C_5_H_9_	4	5.2 ± 0.2
(*RS*)-**3i**	C(CH_3_)_3_	8	6.4 ± 0.2
(*RS*)-**3l**	1-methylindol-2-yl	>32	87.6 ± 0.9
(*RS*)-**3m**	benzofur-2-yl	>32	48.8 ± 0.5
(*RS*)-**3n**	benzothiophen-2-yl	>32	111.6 ± 0.9
(*RS*)-**3o**	benzothiazol-2-yl	>32	66.9 ± 0.5

* Data obtained from earlier work for comparison [27].

**Figure 4:**
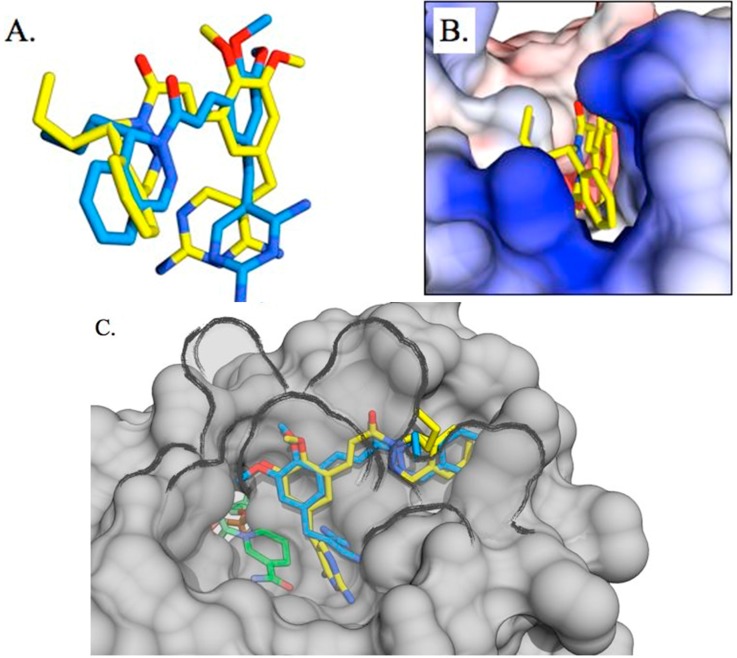
The inhibitor series adopts a low energy conformation prior to binding, and retains this conformation when buried in the DHFR substrate-binding pocket. (**A**) Superposition of (*S*)-**3d** [(*S*)-RAB1] from single crystal analysis (cyan) with that observed when bound by the BaDHFR protein (yellow). Note the highly similar conformation, with differences in torsional rotation to optimize the DAP ring placement, and the induced bend of the dihydrophthalazine from planar (cyan) to 107° to 117° (yellow), critical for interactions with the protein site [6]; (**B**) View of (*S*)-RAB1 bound in the BaDHFR binding site, with the protein van der Waals surface colored by electrostatic potential; (**C**) Highly buried position of (*S*)-RAB1 in the BaDHFR binding site, with part of the protein van der Waals surface shown by outline to permit visualization of the inhibitor (NADPH also visible, green). Note the single crystal (cyan) version of (*S*)-RAB1 adopts a highly similar orientation even in the absence of the DHFR protein. Both (B) and (C) illustrate the limited volume for R^1^ modifications, with favorable occupancy by up to one ring structure, but extension beyond this results in protrusion into the solvent region.

Comparison of the single crystal X-ray structure of (*S*)-**3d** (Figure 3A) with its pose in the DHFR binding site (Figure 4A) shows a very similar conformation. Thus, the enzyme permits the substrate to adopt a low energy conformation when bound with the protein, similar to its preferred solid-state minimum. The R^1^ group at the C-1 dihydrophthalazine stereocenter serves as an interface between the substrate-binding pocket and the surrounding solvent. Figure 4B,C demonstrate the position of the R^1^ group of (*S*)-**3d** ((*S*)-RAB1) within the binding pocket of the *B. anthracis* dihydrofolate reductase (BaDHFR) protein and its proximity to the surrounding medium.

Earlier studies indicated that R^1^ interacts effectively with surrounding amino acid residues in *B. anthracis* DHFR such as Lys33, Thr35, Leu41, Pro56, and Arg58 [15,27]. Derivatives with smaller groups at R^1^, such as (*RS*)-**3a**–**h**, adopted planar conformations within the binding pocket that have more optimal interactions with the guanidinium moiety of Arg58, and hence we observed increased activity for these compounds. Additionally, compounds containing alkenes (*RS*)-**3a-c** have a restricted motion within the pocket, and this also leads to higher activity.

Larger aryl groups at the R^1^ position were previously prepared and studied by Nammalwar and co-workers [13,15]. It was surmised that aryl substituents protruded into solvent space outside the binding pocket, resulting in lesser inhibitory properties. A similar situation was presumed for compounds **3l-o**. These derivatives exhibited lower potency and this was attributed to the larger size and the spatial orientation of R^1^ groups, which forced the molecules to move outside the binding pocket. These observations demonstrated that the protrusion of more hydrophobic R^1^ groups beyond the confines of the protein binding site should be energetically unfavorable and render these compounds as weaker inhibitors.

## 3. Experimental Section

### 3.1. General Information

Commercial anhydrous *N*,*N*-dimethylformamide (DMF) and dimethylsulfoxide (DMSO) were stored under dry nitrogen and transferred by syringe into reactions when needed. Tetrahydrofuran (THF) was dried over potassium hydroxide pellets and distilled from lithium aluminium hydride prior to use. Other commercial reagents [bismuth(III) triflate (Bi(OTf)_3_), butyllithium (*n- and t*-BuLi), magnesium sulfate (MgSO_4_), triethylamine (TEA)] and solvents (acetonitrile (MeCN), dichloromethane (DCM), ethyl acetate (EtOAc), hexanes, methanol (MeOH)) were used as received. Aqueous wash solutions (saturated sodium chloride (NaCl) and ammonium chloride (NH_4_Cl)) were used in work-up procedures.

All reactions were run under dry nitrogen in oven-dried glassware. Reactions were monitored by thin layer chromatography (TLC) on silica gel GF plates (Analtech, No. 21521, Newark, DE, USA) and visualized using a hand-held UV lamp. Preparative column chromatography was carried out on 63–200 µm silica gel (Sorbent Technologies, Norcross, GA, USA). Melting points were determined using a 200 W MEL-TEMP apparatus (Cambridge, MA, USA) and were uncorrected. FT-IR spectra were run as DCM solutions between NaCl disks. Unless otherwise indicated, ^1^H and ^13^C-NMR spectra were measured at 400 MHz and 100 MHz, respectively, in the indicated solvent. Chemical shifts (δ) are referenced to internal (CH_3_)_4_Si and coupling constants (*J*) are given in Hz. Elemental analyses (±0.4%) were performed by Atlantic Microlabs, Inc. (Norcross, GA, USA).

### 3.2. 2,4-Diamino-5-(5-iodo-3,4-dimethoxybenzyl)pyrimidine (**1**)

This compound was prepared from 5-iodovanillin in 70% yield on a 0.40-mol scale according to the literature procedure [15], mp 217–218 °C (lit. [15] mp 217–218 °C). The spectral data matched those reported [15].

### 3.3. Preparation of Phthalazin-2(1H)-yl]-2-propen-1-ones **2a**–**r**

*(±)-1-[1-(2-Methyl-1-propen-1-yl)phthalazin-2(1H)-yl]-2-propen-1-one* (**2a**). This compound was prepared from **4** and 2-methyl-1-propenylmagnesium bromide in 62% yield on a 15.4-mmol scale according to the literature procedure [15]. The spectral data matched those reported [15].

*(±)-1-(1-Allylphthalazin-2(1H)-yl)-2-propen-1-one* (**2b**). A stirred solution of **4** (2.50 g, 19.2 mmol) in dry THF (60 mL) was treated dropwise with a solution of allylmagnesium bromide (12.4 mL, 24.9 mmol, 2 M in THF) for 30 min at 0 °C. The reaction mixture was warmed to room temperature and stirred for 1 h. After completion, the reaction mixture was treated with saturated NH_4_Cl (50 mL) and extracted with EtOAc (3 × 100 mL). The combined organic extracts were washed with saturated NaCl (50 mL), dried (MgSO_4_), filtered, and concentrated under vacuum to give **3b** as a dark brown, viscous liquid. The crude **5b** was dissolved in DCM (70 mL) and TEA (5.87 mL, 41.7 mmol) was added, followed by dropwise addition of acryloyl chloride (1.35 mL, 16.7 mmol) at 0 °C. The reaction mixture was stirred at this temperature for 2 h. The mixture was then treated with saturated NaCl (100 mL), the organic layer was separated, and the aqueous layer was extracted with DCM (2 × 50 mL). The combined extracts were washed with saturated NaCl (50 mL), dried (MgSO_4_), filtered, and concentrated to afford the crude product. This material was purified on a silica gel column eluted with hexanes–EtOAc (7:3) to afford **2b** (2.47 g, 57%) as a viscous, yellow liquid. IR: 1662 cm^−1^; ^1^H-NMR (CDCl_3_): δ 7.60 (s, 1H), 7.43 (td, *J* = 7.4, 1.4 Hz, 1H), 7.35 (td, *J* = 7.6, 1.2 Hz, 1H), 7.30 (dd, *J* = 17.1, 10.5 Hz, 1H), 7.28 (d, *J* = 7.6, 0.6 Hz, 1H), 7.15 (dd, *J* = 7.6, 0.6 Hz, 1H), 6.42 (dd, *J* = 17.1, 2.1 Hz, 1H), 5.91 (t, *J* = 6.4 Hz, 1H), 5.78 (dd, *J* = 10.5, 2.1 Hz, 1H), 5.68 (ddt, *J* = 17.1, 10.1, 7.3 Hz, 1H), 4.98 (dm, *J* = 10.2 Hz, 1H), 4.90 (dm, *J* = 17.0 Hz, 1H), 2.41 (m, 2H); ^13^C-NMR (CDCl_3_): δ 166.3, 142.2, 133.2, 132.7, 131.4, 128.4, 128.1, 127.1, 126.6, 125.6, 123.9, 118.7, 51.1, 39.8.

*(±)-1-(1-Vinylphthalazin-2(1H)-yl)-2-propen-1-one* (**2c**). This compound was prepared via the procedure described for **2b** using **4** (2.50 g, 19.2 mmol) and vinylmagnesium bromide (11.5 mL, 23.0 mmol, 2.0 M in THF) in dry THF (60 mL) to afford **3c**, followed by acylation using TEA (5.3 mL, 37.8 mmol) and acryloyl chloride (1.20 mL, 15.1 mmol) in DCM (70 mL) to give **2c** (2.36 g, 58%) as a colorless, viscous liquid. IR: 1665 cm^−1^; ^1^H-NMR (CDCl_3_): δ 7.59 (s, 1H), 7.45 (td, *J* = 7.4, 0.8 Hz, 1H), 7.40–7.28 (m, 3H), 7.21 (dd, *J* = 7.6, 0.4 Hz, 1H), 6.51 (dd, *J* = 17.4, 2.1 Hz, 1H), 6.37 (d, *J* = 4.9 Hz, 1H), 5.81 (m, 2H), 5.10 (dd, *J* = 10.3, 0.4 Hz, 1H), 4.86 (d, *J* = 17.4 Hz, 1H); ^13^C-NMR (CDCl_3_): δ 166.2, 141.7, 134.7, 131.9, 131.7, 128.8, 128.4, 126.9, 126.8, 125.9, 123.8, 116.2, 53.0.

*(±)-1-(1-Propylphthalazin-2(1H)-yl)-2-propen-1-one* (**2d**). This compound was prepared in 70% yield from **4** (2.00 g, 15.4 mmol) and propylmagnesium chloride (8.45 mL, 16.9 mmol, 2.0 M in ether) in dry THF (50 mL) to afford **5d**, followed by acylation using TEA (1.82 g, 2.50 mL, 18.0 mmol) and acryloyl chloride (1.40 g, 1.26 mL, 15.5 mmol) in DCM (70 mL) to afford **2d** according to the literature procedure [15]. The spectral data matched those reported [15].

*(±)-1-(1-Pentylphthalazin-2(1H)-yl)-2-propen-1-one* (**2e**). This compound was prepared via the procedure described for **2b** from **4** (2.50 g, 19.2 mmol) and *n*-pentylmagnesium bromide (18.4 mL, 18.4 mmol, 1 M in THF) in dry THF (60 mL) to afford **5e**, followed by acylation using TEA (3.27 g, 4.51 mL, 32.4 mmol) and acryloyl chloride (1.05 mL, 12.9 mmol) in DCM (70 mL) to give **2e** (3.05 g, 62%) as an off-white solid, mp 52–54 °C. IR: 1664 cm^−1^; ^1^H-NMR (CDCl_3_): δ 7.61 (s, 1H), 7.42 (td, *J* = 7.4, 1.1 Hz, 1H), 7.33 (m, 2H), 7.26 (d, *J* = 7.0 Hz, 1H), 7.15 (dd, *J* = 7.4, 0.7 Hz, 1H), 6.47 (dd, *J* = 17.2, 2.4 Hz, 1H), 5.83 (t, *J* = 6.7 Hz, 1H), 5.77 (dd, *J* = 10.5, 2.4 Hz, 1H), 1.63 (m, 2H), 1.21 (m, 6H), 0.81 (t, *J* = 6.6 Hz, 3H); ^13^C-NMR (CDCl_3_): δ 166.1, 142.3, 134.0, 131.3, 128.2, 127.9, 127.1, 126.4, 125.6, 123.8, 51.2, 34.9, 31.4, 24.4, 22.3, 13.8.

*(±)-1-(1-Cyclopropylphthalazin-2(1H)-yl)-2-propen-1-one* (**2f**). This compound was prepared in 87% yield from **4** (2.00 g, 15.4 mmol) and cyclopropylmagnesium chloride (33.8 mL, 16.9 mmol, 0.5 M in THF) in dry THF (50 mL) to give **5f**, followed by acylation using TEA (1.86 g, 2.56 mL, 18.4 mmol) and acryloyl chloride (1.39 g, 1.25 mL, 15.4 mmol) in DCM (70 mL) to afford **2f** according to the literature procedure [13]. The spectral data matched those reported [13].

*(±)-1-(1-Cyclobutylphthalazin-2(1H)-yl)-2-propen-1-one* (**2g**). This compound was prepared via the procedure described for **2b** using **4** (2.50 g, 19.2 mmol) and cyclobutylmagnesium bromide (24.9 mL, 24.9 mmol, 1 M in THF) in dry THF (60 mL) to give **5g**, followed by acylation using TEA (3.25 g, 4.48 mL, 32.2 mmol) and acryloyl chloride (1.16 g, 1.04 mL, 12.8 mmol) in DCM (70 mL) to afford **2g** (3.04 g, 66%) as a yellow liquid. IR: 1663 cm^−1^; ^1^H-NMR (CDCl_3_): δ 7.61 (s, 1H), 7.44 (td, *J* = 7.4, 1.5 Hz, 1H), 7.35 (td, *J* = 7.4, 1.5 Hz, 1H), 7.31 (dd, *J* = 17.2, 10.5 Hz, 1H), 7.29 (m, 1H), 7.16 (dd, *J* = 7.5, 0.7 Hz, 1H), 6.48 (dd, *J* = 17.2, 2.0 Hz, 1H), 5.83 (d, *J* = 8.4 Hz, 1H), 5.77 (dd, *J* = 10.5, 2.0 Hz, 1H), 2.60 (sextet, *J* = 8.4 Hz, 1H), 1.98 (m, 1H), 1.90–1.73 (m, 2H), 1.67 (m, 3H); ^13^C-NMR (CDCl_3_): δ 166.5, 143.8, 132.6, 131.3, 128.2, 128.0, 127.1, 126.5, 125.6, 124.0, 54.5, 40.2, 25.6, 24.9, 17.6.

*(±)-1-(1-Cyclopentylphthalazin-2(1H)-yl)-2-propen-1-one* (**2h**). This compound was prepared via the procedure described for **2b** from **4** (2.50 g, 19.2 mmol) and cyclopentylmagnesium bromide (24.9 mL, 24.9 mmol, 1 M in THF) in dry THF (60 mL) to give **5h**, followed by acylation using TEA (3.78 g, 5.20 mL, 37.4 mmol) and acryloyl chloride (1.35 g, 1.21 mL, 14.9 mmol) in DCM (70 mL) to afford **2h** (2.73 g, 56%) as a viscous, colorless liquid. IR: 1663 cm^−1^; ^1^H-NMR (CDCl_3_): δ 7.69 (s, 1H), 7.43 (td, *J* = 7.4, 1.4 Hz, 1H), 7.35 (td, *J* = 7.4, 1.4 Hz, 1H), 7.32 (dd, *J* = 17.2, 10.5 Hz, 1H), 7.28 (dd, *J* = 7.4, 1.4 Hz, 1H), 7.18 (dd, *J* = 7.4, 0.5 Hz, 1H), 6.46 (dd, *J* = 17.2, 2.1 Hz, 1H), 5.80 (d, *J* = 8.6 Hz, 1H), 5.77 (dd, *J* = 10.5, 2.1 Hz, 1H), 2.12 (m, 1H), 1.61 (m, 3H), 1.42 (m, 4H), 1.23 (m, 1H); ^13^C-NMR (CDCl_3_): δ 166.3, 143.6, 133.5, 131.2, 128.3, 128.0, 127.1, 126.8, 125.4, 124.2, 54.1, 45.2, 29.3, 28.7, 24.3, 24.2.

*(±)-1-(1-(tert-Butyl)phthalazin-2(1H)-yl)-2-propen-1-one* (**2i**). A stirred solution of **4** (2.00 g, 17.6 mmol) in 50 mL of dry THF was treated dropwise with a solution of *t*-BuLi (16.9 mL, 16.9 mmol, 1.0 M in heptanes) over a period of 15 min at −78 °C. The reaction was stirred at this temperature for 1 h and then slowly warmed to room temperature and stirred for an additional 30 min. The reaction mixture was added to cold saturated NH_4_Cl (50 mL) and extracted with EtOAc (3 × 50 mL). The combined organic extracts were washed with saturated NaCl (50 mL), dried (MgSO_4_), filtered, and concentrated under vacuum to give **5i** as a dark brown liquid. The crude product was dissolved in DCM (100 mL), and TEA (4.00 g, 5.50 mL, 39.6 mmol) was added, followed by dropwise addition of acryloyl chloride (1.44 g, 1.29 mL, 15.9 mmol) at 0 °C. The reaction mixture was stirred at this temperature for 2 h. The mixture was then added to saturated NaCl (100 mL), the organic layer was separated, and the aqueous layer was extracted with DCM (2 × 50 mL). The combined extracts were washed with saturated NaCl (50 mL), dried (MgSO_4_), filtered, and concentrated to afford the crude product. The crude product was purified on a silica gel column eluted with hexanes–EtOAc (7:3) to afford **2i** as a viscous, yellow liquid (2.79 g, 75%). IR: 1666 cm^−1^; ^1^H-NMR (CDCl_3_): δ 7.65 (s, 1H), 7.46 (td, *J* = 7.4, 1.4 Hz, 1H), 7.37 (td, *J* = 7.4, 1.4 Hz, 1H), 7.34 (dd, *J* = 17.1, 10.1 Hz, 1H), 7.29 (dd, *J* = 7.4, 0.8 Hz, 1H), 7.19 (d, *J* = 7.8 Hz, 1H), 6.43 (dd, *J* = 17.1, 2.3 Hz, 1H), 5.78 (s, 1H), 5.75 (dd, *J* = 10.1, 2.3 Hz, 1H), 0.86 (s, 9H); ^13^C-NMR (CDCl_3_): δ 167.0, 144.6, 133.4, 131.0, 130.6, 128.6, 128.2, 128.0, 127.4, 125.4, 125.1, 58.1, 39.4, 26.6, 25.5.

*(±)-1-(1-(Furan-2-yl)phthalazin-2(1H)-yl)-2-propen-1-one* (**2j**). This compound was prepared in 72% yield from **4** (2.00 g, 15.4 mmol), furan-2-yllithium [from furan (1.20 g, 17.6 mmol) and *n*-BuLi (7.30 mL, 18.3 mmol, 2.5 M in hexanes)] in dry THF (50 mL) to give **5j**, followed by acylation using TEA (2.37 g, 3.26 mL, 23.5 mmol) and acryloyl chloride (1.59 g, 1.43 mL, 17.6 mmol) in DCM (70 mL) to afford **2j** according to the literature procedure [13]. The spectral data matched those reported [13].

*(±)-1-[1-(Thiophen-2-yl)phthalazin-2(1H)-yl]-2-propen-1-one* (**2k**). This compound was prepared in 60% yield from **4** (2.00 g, 15.4 mmol), thiophen-2-ylmagnesium bromide [from 2-bromothiophene (1.77 g, 1.69 mL, 21.0 mmol) and magnesium (0.69 g, 28.4 mmol)] in dry THF (50 mL) to give **5k**. The crude **5k** was acylated using TEA (2.80 g, 3.86 mL, 27.7 mmol) and acryloyl chloride (1.90 g, 1.71 mL, 21.0 mmol) in DCM (70 mL) to afford **2k** according to the literature procedure [13]. The spectral data matched those reported [13].

*(±)-1-[1-(1-Methyl-1H-indol-2-yl)phthalazin-2(1H)-yl]-2-propen-1-one* (**2l**). To a stirred solution of 1-methylindole (1.50 g, 11.4 mmol) in dry THF (25 mL) was added dropwise *n*-BuLi (6.86 mL, 17.2 mmol, 2.5 M in hexanes) over a period of 30 min at −78 °C. The solution was warmed to −25 °C, and stirring was continued at this temperature for 1 h. The reaction mixture was cooled to −78 °C, and a solution of **4** (1.48 g, 11.4 mmol) in dry THF (20 mL) was added dropwise over 30 min. The reaction mixture was stirred at this temperature for 2 h. The mixture was poured into saturated NH_4_Cl (100 mL) and extracted with EtOAc (3 × 50 mL). The combined organic extracts were washed with saturated NaCl (50 mL), dried (MgSO_4_), filtered, and concentrated under vacuum to give **5l** as a light yellow liquid. The crude product **5l** was dissolved in DCM (100 mL), and TEA (2.08 g, 2.87 mL, 20.6 mmol) was added, followed by dropwise addition of acryloyl chloride (0.81 g, 0.73 mL, 8.95 mmol) at 0 °C. The reaction mixture was stirred at this temperature for an additional 2 h. The aqueous layer was added to saturated NaCl (50 mL), and the organic layer was separated. The aqueous layer was extracted with DCM (2 × 30 mL), and the combined organic extracts were washed with saturated NaCl (50 mL), dried (MgSO_4_), filtered, and concentrated to afford the crude product. The product was purified on a silica gel column eluted with hexanes–EtOAc (7:3) to afford **2l** (3.01 g, 62%) as a light yellow solid, mp 69–71 °C. IR: 1657 cm^−1^; ^1^H-NMR (CDCl_3_): δ 7.79 (s, 1H), 7.42–7.34 (complex, 5H), 7.22 (dd, *J* = 17.2, 10.5 Hz, 1H), 7.18 (s, 1H), 7.16 (m, 2H), 7.00 (t, *J* = 7.6 Hz, 1H), 6.45 (dd, *J* = 7.2, 2.0 Hz, 1H), 5.89 (s, 1H), 5.75 (dd, *J* = 10.5, 2.0 Hz, 1H), 3.99 (s, 3H); ^13^C-NMR (CDCl_3_): δ 166.0, 143.3, 138.9, 137.4, 132.3, 132.1, 129.0, 128.6, 127.2, 127.0, 126.7, 126.0, 123.7, 122.0, 120.5, 119.6, 109.6, 103.3, 46.6, 30.6.

*(±)-1-[1-(Benzofuran-2-yl)phthalazin-2(1H)-yl]-2-propen-1-one* (**2m**). The compound was prepared using benzofuran-2-yllithium [from benzofuran (2.00 g, 16.9 mmol) and *n*-BuLi (6.8 mL, 17.0 mmol, 2.5 M in hexanes)] and **4** (2.20 g, 16.9 mmol) in dry THF (75 mL) to give **5m**. The crude **5m** was acylated using TEA (3.05 g, 4.20 mL, 30.2 mmol) and acryloyl chloride (1.09 g, 0.97 mL, 12.0 mmol) in DCM (120 mL) to afford **2m** (2.37 g, 51%) as a light yellow solid, mp 55–57 °C. IR: 1657 cm^−1^; ^1^H-NMR (CDCl_3_): δ 7.84 (d, *J* = 7.8 Hz, 1H), 7.63 (dd, *J* = 7.8, 0.6 Hz, 1H), 7.56 (d, *J* = 8.2 Hz, 1H), 7.48–7.40 (complex, 3H), 7.36 (td, *J* = 7.5, 1.2 Hz, 2H), 7.27 (t, *J* = 7.5 Hz, 1H), 7.23 (s, 1H), 7.23 (d, *J* = 7.5 Hz, 1H), 6.54 (dd, *J* = 17.1, 2.1 Hz, 1H), 5.84 (dd, *J* = 10.4, 2.0 Hz, 1H), 5.02 (s, 1H); ^13^C-NMR (CDCl_3_): δ 166.4, 154.9, 151.1, 141.8, 131.7, 131.0, 139.1, 138.1, 137.7, 126.5, 126.3, 126.0, 125.4, 123.6, 123.3, 121.5, 111.5, 108.0, 42.0.

*(±)-1-[1-(Benzo[b]thiophen-2-yl)phthalazin-2(1H)-yl]-2-propen-1-one* (**2n**). The compound was prepared using benzothiophen-2-yllithium [from benzothiophene (2.00 g, 14.9 mmol) and *n*-BuLi (6.0 mL, 15.0 mmol, 2.5 M in hexanes)] and **4** (1.94 g, 14.9 mmol) in dry THF (75 mL) to give **5n**. The crude **5n** was acylated using TEA (2.86 g, 3.94 mL, 28.3 mmol) and acryloyl chloride (1.02 g, 0.92 mL, 11.3 mmol) in DCM (120 mL) to afford **2n** (2.94 g, 62%) as a light yellow solid, mp 69–71 °C. IR: 1663 cm^−1^; ^1^H-NMR (CDCl_3_): δ 7.86 (m, 1H), 7.81 (m, 2H), 7.70 (s, 1H), 7.51 (t, *J* = 7.6 Hz, 1H), 7.46–7.36 (complex, 5H), 7.32 (d, *J* = 7.2 Hz, 1H), 6.54 (dd, *J* = 17.2, 1.8 Hz, 1H), 5.87 (dd, *J* = 10.3, 1.8 Hz, 1H), 5.05 (s, 1H); ^13^C-NMR (CDCl_3_): δ 166.4, 146.4, 139.8, 139.7, 139.0, 131.8, 131.7, 129.2, 128.2, 126.6, 126.1, 125.8 (2C), 125.7, 124.6, 124.4, 124.2, 122.2, 42.2.

*(±)-1-[1-(Benzo[d]thiazol-2-yl)phthalazin-2(1H)-yl]-2-propen-1-one* (**2o**). The compound was prepared using benzothiazol-2-yllithium [from (2.00 g, 14.8 mmol) and *n*-BuLi (6.5 mL, 16.3 mmol, 2.5 M in hexanes)] and **4** (1.92 g, 14.8 mmol) in dry THF (75 mL) to give **5o**. The crude **5o** was acylated using TEA (2.86 g, 3.94 mL, 28.3 mmol) and acryloyl chloride (1.02 g, 0.92 mL, 11.3 mmol) in DCM (120 mL) to afford **5o** (3.20 g, 68%) as a light yellow solid, mp 68–70 °C. IR: 1663 cm^−1^; ^1^H-NMR (CDCl_3_): δ 7.93 (d, *J* = 8.2 Hz, 1H), 7.75 (dt, *J* = 8.0, 0.6 Hz, 1H), 7.70 (s, 1H), 7.56 (d, *J* = 7.6 Hz, 1H), 7.51 (td, *J* = 7.4, 1.4 Hz, 1H), 7.45–7.27 (complex, 6H), 6.56 (dd, *J* = 17.4, 2.0 Hz, 1H), 5.87 (dd, *J* = 10.5, 2.0 Hz, 1H); ^13^C-NMR (CDCl_3_): δ 169.5, 166.6, 153.0, 141.5, 135.2, 132.2, 130.3, 129.9, 129.3, 127.7, 126.5, 126.3, 125.9, 125.2, 123.6, 123.2, 121.5, 53.2.

*(±)-t-Butyl 2-(2-acryloylphthalazin-2(1H)-yl)acetate* (**2p**). This compound was prepared in 87% yield from *tert*-butyl acetate (2.67 g, 3.08 mL, 23.0 mmol), *n*-BuLi (7.7 mL, 19.3 mmol, 2.5 M in hexanes), **4** (2.99 g, 23.0 mmol), TEA (1.86 g, 2.56 mL, 18.4 mmol) and acryloyl chloride (1.39 g, 1.25 mL, 15.4 mmol) according to the literature procedure [13]. The spectral data matched those reported [13].

*(±)-2-(2-Acryloylpthalazin-2(1H)-yl)acetic acid* (**6**). This compound was prepared in 94% yield from **2p** (1.50 g, 5.00 mmol), and Bi(OTf)_3_ (0.164 g, 0.25 mmol, 5 mol %) in benzene (25 mL) according to the literature procedure [13]. The spectral data matched those reported [13].

*(±)-Ethyl 2-(2-acryloylphthalazin-2(1H)-yl)acetate* (**2q**). This compound was prepared in 95% yield from **6** (1.00 g, 4.10 mmol), and Bi(OTf)_3_ (0.134 g, 0.20 mmol, 5 mol %) in ethanol (25 mL) according to the literature procedure [13]. The spectral data matched those reported [13].

*(±)-Methyl 2-(2-acryloylphthalazin-2(1H)-yl)acetate* (**2r**). This compound was prepared in 95% yield from **6** (1.00 g, 4.10 mmol), Bi(OTf)_3_ (0.134 g, 0.20 mmol, 5 mol %) in CH_3_OH (25 mL) according to the literature procedure [13]. The spectral data matched those reported [13].

### 3.4. Preparation of Drug Candidates **3a**–**r**

*(±)-(E)-3-{5-[(2,4-Diaminopyrimidin-5-yl)methyl]-2,3-dimethoxyphenyl}-1-[1-(2-methyl-1-propen-1-yl)phthalazin-2(1H)yl]-2-propen-1-one* (**3a**). To a stirred solution of **1** (1.00 g, 2.59 mmol) in dry DMF (10 mL) was added a solution of **2a** (0.684 g, 2.85 mmol) in DMF (2 mL), followed by TEA (0.313 g, 0.431 mL, 3.10 mmol), and the Pd pincer-II catalyst (1 mg, 0.0014 mmol). The reaction was heated at 90 °C for 8 h and then cooled using an ice bath. The product was purified by directly pouring the crude reaction mixture onto a 50 cm × 2.5 cm silica gel chromatography column slurry packed with DCM. Impurities were eluted using DCM, and the final product was collected using DCM/MeOH/TEA (95:4:1). Evaporation of the solvent gave a yellow solid, which was dried under high vacuum for 2 h. MeOH (5 mL) was added to dissolve the crude product, followed by ether (10 mL), and the mixture was cooled for 4 h to crystallize the product. The product was filtered and dried under vacuum to afford **3a** as a yellow solid (1.18, 88%). The melting point and spectral data matched those in the literature [15].

*(±)-(E)-1-(1-Allylphthalazin-2(1H)-yl)-3-{5-[(2,4-diaminopyrimidin-5-yl)methyl]-2,3-dimethoxy-phenyl}-2-propen-1-one* (**3b**). This compound was prepared as above using **1** (2.00 g, 5.18 mmol), **2b** (1.55 g, 6.73 mmol), TEA (0.680 g, 0.937 mL, 6.73 mmol), and the Pd pincer-II catalyst (2 mg, 0.0028) in dry DMF (15 mL) to give **3b** (2.03 g, 81%) as a white solid, mp 215–217 °C. IR: 3418, 3123, 3122, 1657, 1641, 1598, 1564 cm^−1^; ^1^H-NMR (DMSO-*d*_6_): δ 7.91 (s, 1H), 7.86 (d, *J* = 16.2 Hz, 1H), 7.62 (d, *J* = 16.2 Hz, 1H), 7.60 (s, 1H), 7.56–7.37 (complex, 4H), 7.24 (s, 1H), 6.99 (s, 1H), 6.19 (br s, 2H), 5.95 (t, *J* = 6.3 Hz, 1H), 5.74 (br s, 2H), 5.66 (ddt, *J* = 17.4, 10.5, 6.3 Hz, 1H), 4.94 (dm, *J* = 10.5 Hz, 1H), 4.86 (dm, *J* = 17.4 Hz, 1H), 3.79 (s, 3H), 3.74 (s, 3H), 3.60 (s, 2H), 2.36 (m, 2H); ^13^C-NMR (DMSO-*d*_6_): δ 165.6, 162.3, 162.2, 155.8, 152.5, 146.0, 142.5, 136.6, 133.2, 132.9, 131.7, 128.3, 127.8, 126.6, 126.0, 123.7, 118.4, 118.3, 117.9, 114.8, 105.7, 60.8, 55.7, 54.9, 50.4, 32.4 (1 aromatic/alkene C unresolved); Anal. Calcd for C_27_H_28_N_6_O_3_·1.3 H_2_O: C, 63.84; H, 6.07; N, 16.54. Found: C, 63.84; H, 5.69; N, 16.28.

*(±)-(E)-3-{5-[(2,4-Diaminopyrimidin-5-yl)methyl]-2,3-dimethoxyphenyl}-1-(1-vinylphthalazin-2(1H)-yl)-2-propen-1-one* (**3c**). This compound was prepared as above using **1** (1.60 g, 4.15 mmol), **2c** (1.14 g, 5.38 mmol), TEA (0.543 g, 0.750 mL, 5.38 mmol) and the Pd pincer-II catalyst (1.5 mg, 0.0021 mmol) in dry DMF (15 mL) to give **3c** (1.65 g, 85%) as a white solid, mp 210–212 °C. IR: 3354, 3169, 1638, 1593, 1567 cm^−1^; ^1^H-NMR (DMSO-*d*_6_): δ 7.91 (s, 1H), 7.89 (d, *J* = 16.0 Hz, 1H), 7.68 (d, *J* = 16.0 Hz, 1H), 7.60–7.44 (complex, 5H), 7.32 (d, *J* = 1.3 Hz, 1H), 7.25 (br s, 2H), 7.04 (d, *J* = 1.4, 1H), 6.73 (br s, 2H), 6.40 (d, *J* = 4.5 Hz, 1H), 5.80 (ddd, *J* = 1.5, 10.2, 4.5 Hz, 1H), 5.08 (d, *J* = 10.2 Hz, 1H), 4.78 (d, *J* = 17.0 Hz, 1H), 3.81 (s, 3H), 3.76 (s, 3H), 3.65 (s, 2H); ^13^C-NMR (DMSO-*d*_6_): δ 165.6, 163.2, 157.7, 152.6, 146.8, 146.3, 142.1, 136.9, 135.4, 135.0, 132.0, 131.7, 128.6, 127.9, 126.9, 126.3, 123.5, 118.8, 117.9, 115.3, 115.0, 107.6, 60.8, 55.9, 52.5, 31.9; Anal. Calcd for C_26_H_26_N_6_O_3_·4.6 H_2_O·0.1 C_2_H_5_OH: C, 56.21; H, 5.32; N, 15.01. Found: C, 56.25; H, 4.99; N, 15.04.

*(±)-(E)-3-{5-[(2,4-Diaminopyrimidin-5-yl)methyl]-2,3-dimethoxyphenyl}-1-(1-propylphthalazin-2(1H)-yl)-2-propen-1-one* (**3d**). This compound was prepared as above using **1** (1.00 g, 2.59 mmol), **2d** (0.701 g, 3.10 mmol), TEA (0.313 g, 0.432 mL, 3.10 mmol), and the Pd pincer-II catalyst (1 mg, 0.0014 mmol) in dry DMF (15 mL) to give **3d** (1.08 g, 86%) as a white solid. The melting point and spectral data matched those in the literature [15].

*(±)-(E)-3-{5-[(2,4-Diaminopyrimidin-5-yl)methyl]-2,3-dimethoxyphenyl}-1-(1-pentylphthalazin-2(1H)-yl)-2-propen-1-one* (**3e**). This compound was prepared as above using **1** (1.60 g, 4.15 mmol), **2e** (1.39 g, 5.35 mmol), TEA (0.500 g, 0.689 mL, 4.95 mmol), and the Pd pincer-II catalyst (1.5 mg, 0.0021 mmol) in dry DMF (15 mL) to give **3e** (1.91 g, 90%) as a white solid, mp 212–213 °C. IR: 3363, 3173, 1638, 1590, 1560 cm^−1^; ^1^H-NMR (DMSO-*d*_6_): δ 7.92 (s, 1H), 7.86 (d, *J* = 16.4 Hz, 1H), 7.64 (d, *J* = 16.4 Hz, 1H), 7.54 (s, 1H), 7.52 (m, 2H), 7.43 (td, *J* = 7.4, 0.8 Hz, 1H), 7.38 (d, *J* = 1.6 Hz, 1H), 7.28 (d, *J* = 1.6 Hz, 1H), 7.08 (br s, 2H), 7.02 (d, *J* = 1.5 Hz, 1H), 6.58 (br s, 2H), 5.82 (t, *J* = 6.7 Hz, 1H), 3.79 (s, 3H), 3.74 (s, 3H), 3.63 (s, 2H), 1.53 (m, 2H), 1.16 (m, 6H) 0.77 (t, *J* = 6.6 Hz, 3H); ^13^C-NMR (DMSO-*d*_6_): δ 165.6, 163.1, 158.3, 152.6, 148.2, 146.2, 142.8, 136.5, 135.2, 133.6, 131.7, 128.3, 127.9, 126.5, 126.1, 123.6, 118.7, 118.0, 114.9, 107.3, 60.8, 55.8, 50.5, 34.5, 32.0, 30.9, 24.0, 21.9, 13.8; Anal. Calcd for C_29_H_34_N_6_O_3_·3.9 H_2_O·0.3 C_2_H_5_OH: C, 59.94; H, 6.42; N, 14.21. Found: C, 59.61; H, 6.42; N, 14.21.

*(±)-(E)-1-(1-Cyclopropylphthalazin-2(1H)-yl)-3-{5-[(2,4-diaminopyrimidin-5-yl)methyl]-2,3-dimethoxyphenyl}-2-propen-1-one* (**3f**). This compound was prepared as above using **1** (1.00 g, 2.59 mmol), **2f** (0.645 g, 2.80 mmol), TEA (0.313 g, 0.432 mL, 3.10 mmol), and the Pd pincer-II catalyst (1 mg, 0.0014 mmol) in dry DMF (15 mL) to give **3f** (1.05 g, 84%) as a white solid. The melting point and spectral data matched those in the literature [13].

*(±)-(E)-1-(1-Cyclobutylphthalazin-2(1H)-yl)-3-{5-[(2,4-diaminopyrimidin-5-yl)methyl]-2,3-dimethoxyphenyl}-2-propen-1-one* (**3g**). This compound was prepared as above using **1** (1.50 g, 3.89 mmol), **2g** (1.21 g, 5.04 mmol), TEA (0.510 g, 0.702 mL, 5.05 mmol), and the Pd pincer-II catalyst (1.5 mg, 0.0021 mmol) in dry DMF (15 mL) to give **3g** (1.68 g, 87%) as a white solid, mp 125–127 °C. IR: 3397, 3272, 1642, 1605, 1562 cm^−1^; ^1^H-NMR (DMSO-*d*_6_): δ 7.93 (s, 1H), 7.85 (d, *J* = 16.0 Hz, 1H), 7.64 (d, *J* = 16.0 Hz, 1H), 7.59 (s, 1H), 7.52 (m, 2H), 7.44 (m, 2H), 7.25 (s, 1H), 7.00 (s, 1H), 6.39 (br s, 2H), 5.92 (br s, 2H), 5.86 (d, *J* = 8.2 Hz, 1H), 3.79 (s, 3H), 3.74 (s, 3H), 3.60 (s, 2H), 2.54 (sextet, *J* = 8.2 Hz, 1H), 1.84 (m, 3H), 1.63 (m, 3H); ^13^C-NMR (DMSO-*d*_6_): δ 165.9, 162.4, 161.4, 154.0, 152.5, 146.0, 143.3, 136.6, 136.3, 132.1, 131.7, 128.3, 127.8, 126.6, 126.1, 123.8, 118.4, 117.9, 114.8, 106.1, 60.8, 55.8, 53.8, 32.3, 25.1, 24.2, 17.1; Anal. Calcd for C_28_H_30_N_6_O_3_·1.6 H_2_O: C, 60.27; H, 6.31; N, 14.84. Found: C, 60.08; H, 6.07; N, 15.01.

*(±)-(E)-1-(1-Cyclopentylphthalazin-2(1H)-yl)-3-{5-[(2,4-diaminopyrimidin-5-yl)methyl]-2,3-dimethoxyphenyl}-2-propen-1-one* (**3h**). This compound was prepared as above using **1** (1.50 g, 3.89 mmol), **2h** (1.28 g, 5.04 mmol), TEA (0.510 g, 0.702 mL, 5.05 mmol), and the Pd pincer-II catalyst (1.5 mg, 0.0021 mmol) in dry DMF (15 mL) to give **3h** (1.83 g, 92%) as a white solid, mp 190–192 °C. IR: 3338, 3174, 1637, 1594, 1560 cm^−1^; ^1^H-NMR (DMSO-*d*_6_, 400 MHz): δ 7.96 (s, 1H), 7.81 (d, *J* = 16.1 Hz, 1H), 7.60 (d, *J* = 16.1 Hz, 1H), 7.52 (s, 1H), 7.49 (m, 1H), 7.42 (d, *J* = 7.4 Hz, 2H), 7.38 (d, *J* = 7.4, 1H), 7.23 (d, *J* = 1.6 Hz, 1H), 6.97 (d, *J* = 1.6 Hz, 1H), 6.86 (br s, 2H), 6.37 (br s, 2H), 5.74 (d, *J* = 8.4 Hz, 1H), 3.76 (s, 3H), 3.70 (s, 3H), 3.58 (s, 2H), 2.10 (sextet, *J* = 8.4 Hz, 1H), 1.50 (m, 3H), 1.36 (m, 3H), 1.22 (m, 2H); ^13^C-NMR (DMSO-*d_6_*): δ 165.4, 162.9, 159.2, 152.6, 149.7, 146.1, 144.1, 136.6, 135.5, 133.1, 131.6, 128.3, 127.9, 126.9, 125.9, 124.1, 118.6, 118.0, 114.9, 107.0, 60.8, 55.8, 53.4, 44.6, 32.1, 29.0, 28.2, 24.1, 24.0; Anal. Calcd for C_29_H_32_N_6_O_3_·3.5 H_2_O: C, 60.32; H, 5.84; N, 14.55. Found: C, 60.19; H, 5.79; N, 14.57.

*(±)-(E)-1-(1-tert-Butylphthalazin-2(1H)-yl)-3-{5-[(2,4-diaminopyrimidin-5-yl)methyl]-2,3-dimethoxyphenyl}-2-propen-1-one* (**3i**). This compound was prepared as above using **1** (2.20 g, 5.70 mmol), **2i** (1.77 g, 7.32 mmol), TEA (0.748 g, 1.03 mL, 7.41 mmol), and the Pd pincer-II catalyst (2 mg, 0.0028 mmol) in dry DMF (15 mL) to give **3i** (2.36 g, 83%) as a white solid, mp 228–230 °C. IR: 3354, 3174, 1637, 1590, 1561 cm^−1^; ^1^H-NMR (DMSO-*d*_6_): δ 7.97 (s, 1H), 7.84 (d, *J* = 16.0 Hz, 1H), 7.71 (d, *J* = 16.0 Hz, 1H), 7.56 (td, *J* = 7.5, 1.6 Hz, 1H), 7.53 (m, 2H), 7.48 (td, *J* = 7.4, 1.2 Hz, 1H), 7.46 (br s, 2H), 7.39 (d, *J* = 7.8 Hz, 1H), 7.32 (d, *J* = 1.5 Hz, 1H), 7.02 (d, *J* = 2.0 Hz, 1H), 6.92 (br s, 2H), 5.79 (s, 1H), 3.80 (s, 3H), 3.73 (s, 3H), 3.64 (s, 2H), 0.80 (s, 9H); ^13^C-NMR (DMSO-*d*_6_): δ 166.7, 163.4, 156.8, 152.6, 146.2, 145.1, 144.7, 136.3, 134.6, 131.3, 129.8, 128.5, 128.4, 128.0, 125.5, 125.1, 118.9, 118.4, 114.9, 107.9, 60.8, 53.7, 55.9, 39.0, 31.8, 26.4 (3C); Anal. Calcd for C_28_H_32_N_6_O_3_·2.4 H_2_O·1.1 C_2_H_5_OH: C, 56.50; H, 5.96; N, 14.21. Found: C, 56.31; H, 5.74; N, 13.93.

*(±)-(E)-3-{5-[(2,4-Diaminopyrimidin-5-yl)methyl]-2,3-dimethoxyphenyl}-1-[1-(furan-2-yl)phthalazin-2(1H)-yl]-2-propen-1-one* (**3j**). This compound was prepared as above using **1** (1.00 g, 2.59 mmol), **2j** (0.785 g, 3.12 mmol), TEA (0.313 g, 0.432 mL, 3.10 mmol), and the Pd pincer-II catalyst (1 mg, 0.0014 mmol) in dry DMF (15 mL) to give **3j** (1.19 g, 90%) as a white solid. The melting point and spectral data matched those in the literature [13].

*(±)-(E)-3-{5-[(2,4-Diaminopyrimidin-5-yl)methyl]-2,3-dimethoxyphenyl}-1-[1-(thiophen-2-yl)phthalazin-2(1H)-yl]-2-propen-1-one* (**3k**). This compound was prepared as above using **1** (1.00 g, 2.59 mmol), **2k** (0.836 g, 3.12 mmol), TEA (0.313 g, 0.432 mL, 3.10 mmol), and the Pd pincer-II catalyst (1 mg, 0.0014 mmol) in dry DMF (15 mL) to give **3k** (1.10 g, 81%) as a white solid. The melting point and spectral data matched those in the literature [13].

*(±)-(E)-3-{5-[(2,4-Diaminopyrimidin-5-yl)methyl]-2,3-dimethoxyphenyl}-1-[1-(1-methyl-1H-indol-2-yl)phthalazin-2(1H)-yl]-2-propen-1-one* (**3l**). This compound was prepared as above using **1** (1.30 g, 3.37 mmol), **2l** (1.27 g, 4.04 mmol), TEA (0.440 g, 0.60 mL, 4.36 mmol), and the Pd pincer-II catalyst (1.3 mg, 0.0018 mmol) in dry DMF (15 mL) to give **3l** (1.45 g, 75%) as a white solid, mp 178–180 °C. IR: 3345, 3150, 1634, 1602, 1562 cm^−1^; ^1^H-NMR (DMSO-d_6_): δ 8.12 (s, 1H), 7.86 (d, *J* = 16.0 Hz, 1H), 7.63 (d, *J* = 16.0 Hz, 1H), 7.62 (d, *J* = 7.8 Hz, 1H), 7.55–7.48 (complex, 4H), 7.44 (d, *J* = 7.2 Hz, 1H), 7.36 (d, *J* = 7.8 Hz, 1H), 7.27 (d, *J* = 1.5 Hz, 1H), 7.26 (s, 1H), 7.11 (td, *J* = 7.4, 0.8 Hz, 1H), 7.02 (br s, 2H), 7.01 (d, *J* = 1.5 Hz, 1H), 6.95 (td, *J* = 7.4, 0.8 Hz, 1H), 6.52 (br s, 2H), 5.92 (s, 1H), 4.05 (s, 3H), 3.78 (s, 3H), 3.71 (s, 3H), 3.61 (s, 2H); ^13^C-NMR (DMSO-d_6_): δ 165.2, 163.0, 158.5, 152.6, 148.5, 146.2, 142.8, 140.3, 137.2, 136.6, 135.3, 132.3, 128.7, 127.8, 126.8, 126.7, 123.1, 121.6, 120.0, 119.4, 118.7, 117.8, 115.1, 110.1, 107.2, 100.9, 60.8, 55.8, 46.3, 32.0, 30.5 (2 aromatic C unresolved); Anal. Calcd for C_33_H_31_N_7_O_3_·4.1 H_2_O: C, 61.70; H, 5.28; N, 14.92. Found: C, 61.75; H, 5.13; N, 15.25.

*(±)-(E)-1-[1-(Benzofuran-2-yl)phthalazin-2(1H)-yl]-3-{5-[(2,4-diaminopyrimidin-5-yl)methyl]-2,3-dimethoxyphenyl}-2-propen-1-one* (**3m**). This compound was prepared as above using **1** (1.80 g, 4.66 mmol), **2m** (1.83 g, 6.06 mmol), TEA (0.615 g, 0.85 mL, 6.06 mmol), and the Pd pincer-II catalyst (2 mg, 0.0028 mmol) in dry DMF (15 mL) to give **3m** (2.09 1.51 g, 80%) as a light yellow solid, mp 222–224 °C. IR: 3345, 3150, 1634, 1602, 1562 cm^−1^; ^1^H-NMR (DMSO-*d*_6_): δ 7.83 (d, *J* = 16.0 Hz, 1H), 7.83 (m, 2H), 7.73 (d, *J* = 16.0 Hz, 1H), 7.73 (obscured, 1H), 7.61 (s, 1H), 7.58 (t, *J* = 7.4 Hz, 1H), 7.53 (s, 1H), 7.52–7.42 (complex, 4H), 7.33 (t, *J* = 7.4 Hz, 1H), 7.17 (d, *J* = 1.7 Hz, 1H), 7.05 (d, *J* = 1.7 Hz, 1H), 6.18 (br s, 2H), 5.68 (br s, 2H), 5.05 (s, 1H), 3.78 (s, 3H), 3.76 (s, 3H), 3.60 (s, 2H); ^13^C-NMR (DMSO-*d*_6_): δ 166.1, 162.3, 162.1, 155.5, 154.4, 152.5, 150.5, 146.1, 141.2, 137.6, 136.6, 132.1, 131.3, 128.5, 127.8, 127.7, 126.8, 126.0, 125.7, 123.6, 123.2, 122.1, 119.2, 117.8, 115.2, 111.6, 108.7, 105.8, 60.7, 55.8, 42.0, 32.4; Anal. Calcd for C_32_H_28_N_6_O_4_: C, 68.56; H, 5.03; N, 14.92. Found: C, 68.30; H, 4.95; N, 14.92.

*(±)-(E)-1-[1-(Benzo[b]thiophen-2-yl)phthalazin-2(1H)-yl]-3-{5-[(2,4-diaminopyrimidin-5-yl)methyl]-2,3-dimethoxyphenyl}-2-propen-1-one* (**3n**). This compound was prepared as above using **1** (1.60 g, 4.15 mmol), **2n** (1.71 g, 5.38 mmol), TEA (0.543 g, 0.75 mL, 5.38 mmol), and the Pd pincer-II catalyst (1.5 mg, 0.0021 mmol) in dry DMF (15 mL) to give **3n** (1.86 g, 78%) as a yellow solid, mp 256–258 °C. IR: 3347, 3180, 1637, 1601, 1559 cm^−1^; ^1^H-NMR (DMSO-d_6_): δ 8.27 (d, *J* = 6.4 Hz, 1H), 8.10–7.30 (complex, 10H), 7.15 (s, 2H), 6.18 (br s, 2H), 5.76 (s, 1H), 5.65 (br s, 2H), 5.06 (s, 2H), 3.84 (s, 3H), 3.80 (s, 3H), 3.67 (s, 2H); ^13^C-NMR (DMSO-d_6_): δ 165.9, 162.9, 158.8, 152.6, 149.1, 146.3, 144.5, 142.5, 139.1, 138.5, 137.6, 135.4, 132.4, 131.5, 129.3, 127.7, 127.3, 126.5, 124.6, 124.5, 123.8, 123.5, 122.4 (2C), 118.8, 117.6, 115.1, 107.1, 60.8, 55.8, 49.6, 32.0; Anal. Calcd for C_32_H_28_N_6_O_3_S·2.1 H_2_O: C, 62.55; H, 5.18; N, 13.14. Found: C, 62.72; H, 4.91; N, 13.06.

*(±)-(E)-1-[1-(Benzo[d]thiazol-2-yl)phthalazin-2(1H)-yl]-3-{5-[(2,4-diaminopyrimidin-5-yl)methyl]-2,3-dimethoxyphenyl}-2-propen-1-one* (**3o**). This compound was prepared as above using **1** (1.50 g, 3.89 mmol), **2o** (1.61 g, 5.04 mmol), TEA (0.510 g, 0.70 mL, 5.05 mmol), and the Pd pincer-II catalyst (1 mg, 0.0014 mmol) in dry DMF (15 mL) to give **3o** (1.61 g, 72%) as a white solid, mp 145–147 °C. IR: 3339, 3202, 1660, 1614, 1564 cm^−1^; ^1^H-NMR (DMSO-*d*_6_): δ 8.04 (s, 1H), 8.03 (obscured dm, 1H), 7.95 (d, *J* = 16.0 Hz, 1H), 7.91 (dd, *J* = 7.4, 0.8 Hz, 1H), 7.74 (d, *J* = 16.0 Hz, 1H), 7.63 (td, *J* = 7.4, 1.2 Hz, 1H), 7.60 (m, 2H), 7.54 (td, *J* = 7.4, 0.8 Hz, 1H), 7.47 (td, *J* = 7.4, 0.8 Hz, 1H), 7.45 (s, 1H), 7.40 (td, *J* = 7.4, 1.2 Hz, 1H), 7.34 (d, *J* = 1.6 Hz, 1H), 7.04 (d, *J* = 1.6 Hz, 1H), 6.66 (br s, 2H), 6.17 (br s, 2H), 5.77 (s, 1H), 3.81 (s, 3H), 3.75 (s, 3H), 3.63 (s, 2H); ^13^C-NMR (DMSO-d_6_): δ 170.1, 168.1, 162.6, 160.3, 152.5, 152.2, 151.9, 146.3, 141.9, 137.8, 136.0, 134.7, 132.3, 130.1, 129.5, 127.8, 127.6, 126.7, 126.4, 125.5, 123.2, 122.8, 122.4, 118.6, 117.3, 115.2, 106.5, 60.8, 55.8, 52.5, 32.2; Anal. Calcd for C_31_H_27_N_7_O_3_S·3.0 H_2_O·0.5 C_2_H_5_OH: C, 58.70; H, 5.12; N, 14.08. Found: C, 58.49; H, 4.81; N, 14.15.

*(±)-tert-Butyl (E)-2-[2-(3-{5-[(2,4-diaminopyrimidin-5-yl)methyl]-2,3-dimethoxyphenyl}acryloyl)-1,2-dihydrophthalazin-1-yl]acetate* (**3p**). This compound was prepared as above using **1** (1.00 g, 2.59 mmol), **2p** (0.935 g, 3.12 mmol), TEA (0.313 g, 0.432 mL, 3.10 mmol), and the Pd pincer-II catalyst (1 mg, 0.0014 mmol) in dry DMF (15 mL) to give **3p** (1.20 g, 83%) as a white solid. The melting point and spectral data matched those in the literature [13].

*(±)-Ethyl (E)-2-[2-(3-{5-[(2,4-diaminopyrimidin-5-yl)methyl]-2,3-dimethoxyphenyl}acryloyl)-1,2-dihydrophthalazin-1-yl]acetate* (**3q**). This compound was prepared as above using **1** (1.00 g, 2.59 mmol), **2q** (0.849 g, 3.12 mmol), TEA (0.313 g, 0.432 mL, 3.10 mmol), and the Pd pincer-II catalyst (1 mg, 0.0014 mmol) in dry DMF (15 mL) to give **3q** (1.10 g, 80%) as a white solid. The melting point and spectral data matched those in the literature [13].

*(±)-Methyl (E)-2-[2-(3-{5-[(2,4-diaminopyrimidin-5-yl)methyl]-2,3-dimethoxyphenyl}acryloyl)-1,2-dihydrophthalazin-1-yl-acetate* (**3r**). This compound was prepared as above using **1** (1.00 g, 2.59 mmol), **2r** (0.805 g, 3.12 mmol), TEA (0.313 g, 0.432 mL, 3.10 mmol), and the Pd pincer-II catalyst (1 mg, 0.0014 mmol) in dry DMF (15 mL) to give **3r** (1.10 g, 82%) as a white solid. The melting point and spectral data matched those in the literature [13].

### 3.5. Enantiomer Resolutions for (±)-**3a** and (±)-**3d**

Chiral HPLC enantiomer separations for (±)-**3a** and (±)-**3d** were performed using a Waters 600 series pump in conjunction with a Waters Model 2487 (Milford, MA, USA). The Empower 3 software (Orlando, FL, USA) was used for instrument control, data acquisition, and data analysis. A Chiralpak^®^ IA column (10 mm × 250 mm id) from Chiral Technologies (West Chester, PA, USA) was used for all of the analyses. The mobile phase consisted of MeCN–MeOH–Et_2_NH (50:50:0.1), and the samples were dissolved in the mobile phase. All HPLC separations were performed at 25 °C with 3 mL/min flow rate, while detection was monitored at 230 nm. For semi-preparative analyses, a 5 mL sample with a concentration of 2 mg/mL was injected. In both cases the (*S*) enantiomer eluted first.

### 3.6. X-ray Structure for (S,E)-3-{5-[(2,4-Diaminopyrimidin-5-yl)methyl]-2,3-dimethoxyphenyl}-1-(1-propylphthalazin-2(1H)-yl)-2-propen-1-one [(S)-(**3d**), (S)-RAB1]

A colorless, plate-shaped crystal of dimensions 0.340 × 0.140 × 0.050 mm was selected for structural analysis. Intensity data for this compound were collected using a diffractometer with a Bruker APEX ccd area detector [30,31] and graphite-monochromated Mo Kα radiation (λ = 0.71073 Å). The sample was cooled to 100(2) K. Cell parameters were determined from a non-linear least squares fit of 2159 peaks in the range 2.50° < θ < 24.72°. A total of 18456 data were measured in the range 1.312 < θ < 25.998° using φ and ω oscillation frames. The data were corrected for absorption by the empirical method [32] giving minimum and maximum transmission factors of 0.971 and 0.996. The data were merged to form a set of 9928 independent data with R(int) = 0.0596 and a coverage of 99.9%.

The triclinic space group *P*1 was determined by statistical tests and verified by subsequent refinement. The structure was solved by direct methods and refined by full-matrix least-squares methods on *F*^2^ [33]. The positions of hydrogens bonded to carbons and nitrogens were initially determined by geometry and were refined using a riding model. Hydrogens bonded to oxygens were located on a difference map, and their positions were refined with a riding model. Non-hydrogen atoms were refined with anisotropic displacement parameters. Hydrogen atom displacement parameters were set to 1.2 (1.5 for methyl) times the isotropic equivalent displacement parameters of the bonded atoms. A total of 676 parameters were refined against 9 restraints and 9928 data to give wR(*F*^2^) = 0.1912 and S = 0.968 for weights of w = 1/[σ^2^(*F*^2^) + (0.0960P)^2^], where P = [*F*_o_^2^ + 2*F*_c_^2^]/3. The final R(*F*) was 0.0665 for the 5384 observed, [*F* > 4σ(*F*)], data. The largest shift/s.u. was 0.001 in the final refinement cycle. The final difference map had maxima and minima of 0.486 and −0.417 e/Å^3^, respectively. The absolute structure was determined by refinement of the Flack parameter [34]. The three polar axis restraints were taken from Flack and Schwarzenbach [35].

### 3.7. Biological Potency Measurements

Measurements of the MIC and the K_i_ utilized a racemic mixture, unless otherwise noted, of each compound, as described earlier [8,13,27,36]. In brief, MIC values were based on standardized cultures of *B. anthracis* Sterne strain as prescribed by the CLSI [29]. Evaluation of growth utilized spectrophotometric values of turbidity at 600 nm and on visual inspection for assessment of bacterial growth. The lowest concentration that yielded no growth after 18 h incubation was assigned as the MIC. Evaluation of the enzymatic activity and inhibition utilized purified DHFR protein cloned from *B. anthracis* Sterne strain and expressed recombinantly in *E. coli* BL21 (DE3) cells. The reaction was reconstituted, including the NADPH co-factor, and was initiated by the addition of the dihydrofolate substrate. The reaction was carried out at 30 °C, and the linear rate was monitored for 2.8 min. These rates were plotted as a function of inhibitor concentration, and the 50% activity point was calculated using a 4-parameter curve fit. These IC_50_ values were converted to K_i_ values using the Cheng-Prusoff equation [37].

## 4. Conclusions

A newly developed protocol, employing dichlorobis[1-(dicyclohexylphosphanyl)piperidine]-palladium(II) (Pd pincer-II), allowed for the Heck synthesis of potential drugs **3a**–**r** under milder conditions, which gave higher yields for substrates with sensitive/reactive functional groups. The catalyst was highly active and enabled the reaction to be performed at much lower temperatures and in shorter reaction times with low catalyst loading. Single crystal analysis of the most active enantiomer of the lead compound revealed an orientation in the unit cell highly similar to that observed for this isomer seated in the DHFR protein binding site. This could be a highly favorable property in terms of the energetics of binding, as it seems that this inhibitor requires only limited re-orientation to conform to the optimum position needed for binding to the enzyme substrate site. Biological studies demonstrated that derivatives with smaller groups at R^1^ showed greater activity, with planar groups such as allyl (**3b**) and vinyl (**3c**) exhibiting the highest potency both in inhibition of *B. anthracis* culture growth and in the DHFR enzymatic assay. On the other hand, larger heteroaromatic substituents, such as those in **3l**–**o**, tempered the inhibition of the whole cell growth. These results reinforced previous findings on the role of size and nature of functional groups that could fit into the binding pocket. An in-house separation protocol for resolution of the *S* and *R* enantiomers was developed for both **3a** and **3d**. Screening indicated that the *S* isomers produced greater inhibition at both the whole cell growth and the enzymatic level with the DHFR protein.

## Supplementary Materials

Supplementary materials can be accessed at: http://www.mdpi.com/1420-3049/20/04/7222/s1.

Electronic Supplementary Information (ESI) available: CCDC 1052963 contains supplementary crystallographic data for (*S*)-RAB1 [(*S*)-**3d**], see doi:10.3390/molecules20047222. These data can be obtained free of charge via http://www.ccdc.cam.ac.uk/conts/retrieving.html (or from the CCDC, 12 Union Road, Cambridge CB2 1EZ, UK; Fax: +44 1223 336033; E-mail: deposit@ccdc.cam.ac.uk).
