# Supplementary material for: Evaluation of New Dihydrophthalazine-Appended 2,4-Diaminopyrimidines against Bacillus anthracis: Improved Syntheses Using a New Pincer Complex
Source: Molecules. 2015 Apr 21;20(4):7222–44. doi: 10.3390/molecules20047222 (PMC4445145; doi:10.3390/molecules20047222)
Supplement: Supplementary file 1 [file molecules-20-07222-s001.pdf]

Supplementary Materials

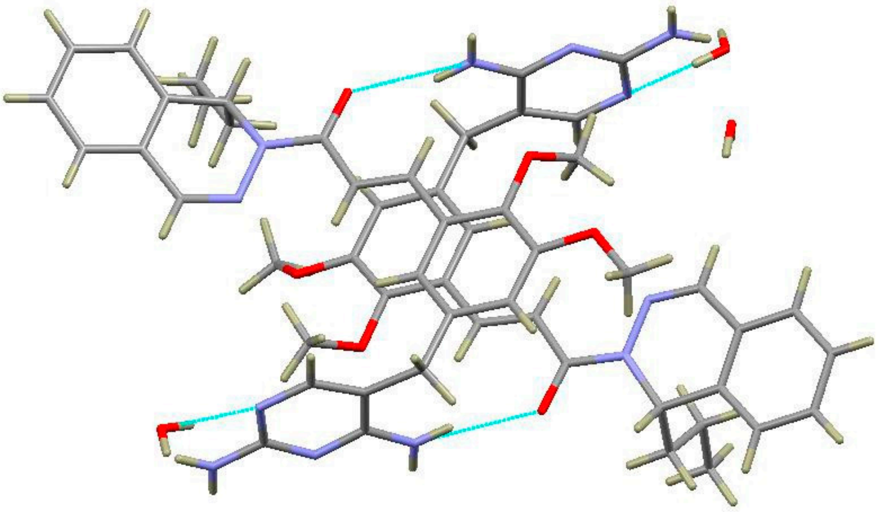

Figure S1. Unit Cell.

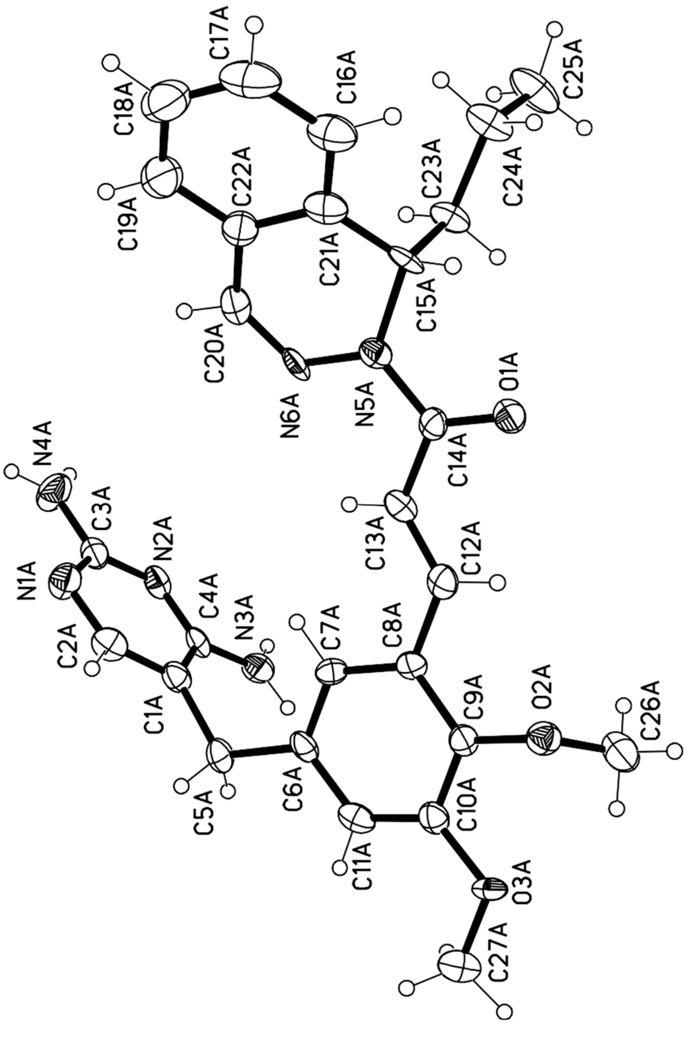

(A)

Figure S2. *Cont.*

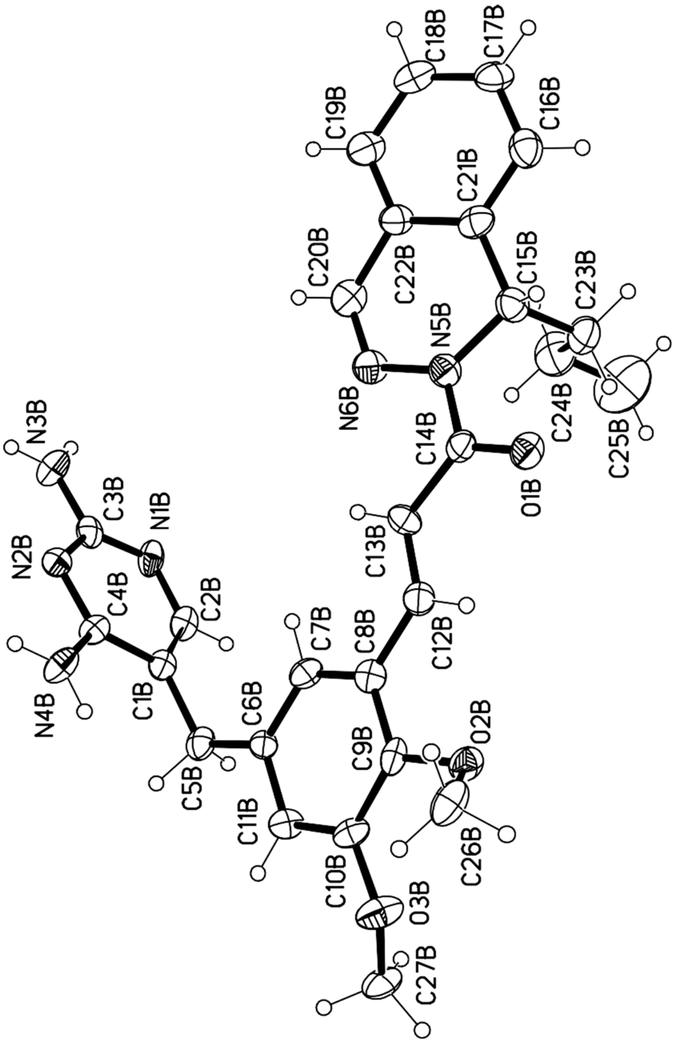

(**B**)

Figure S2. (**A**) Thermal Ellipsoid Plot of Molecule 1; (**B**) Thermal Ellipsoid Plot of Molecule 2.

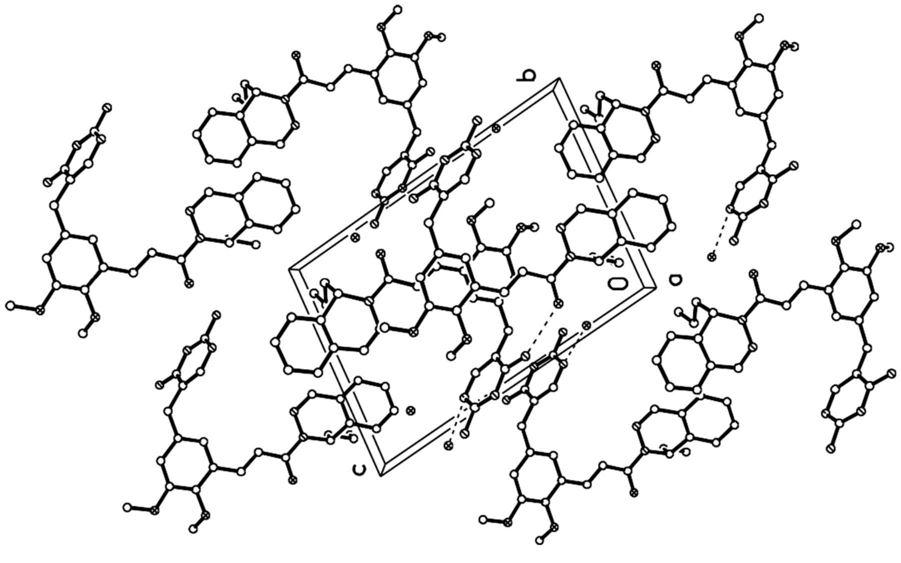

Figure S3. Packing Diagram.

Comment

The unit cell contained two molecules of interest and 2.5 water molecules. Restraints on the displacement parameters of one water were required. The displacement ellipsoids were drawn at the 50% probability level.

Table S1. Crystal data and structure refinement for SSB-01-13F1.

**Crystal Parameter**	**Crystal data**
Empirical formula	$(C_{27}H_{30}N_6O_3)$ 1.25(H_2O) $C_{27}H_{32.5}N_6O_{4.25}$
Formula weight	509.09
Crystal system	triclinic
Space group	*P*1
Unit cell dimensions	*a* = 7.9712(19) Å; α = 98.827(5)°<br>*b* = 10.954(3) Å; β = 102.608(5)°<br>*c* = 16.178(4) Å; γ = 96.072(4)°
Volume	1347.9(6) $Å^3$
Z, Z'	2, 2
Density (calculated)	1.254 Mg/m^3
Wavelength	0.71073 Å
Temperature	100(2) K
F(000)	541
Absorption coefficient	0.087 mm^{-1}
Absorption correction	semi-empirical from equivalents
Max. and min. transmission	0.996 and 0.971
Theta range for data collection	1.312 to 25.998°
Reflections collected	18456
Independent reflections	9928 [R(int) = 0.0596]
Data / restraints / parameters	9928 / 9 / 676
$wR(F^2$ all data)	$wR2$ = 0.1912
$R(F$ obsd data)	$R1$ = 0.0665
Goodness-of−Fit on F^2	0.968
Observed data [I > 2 (I)]	5384
Absolute structure parameter	−2.2(10)
Largest and mean shift / s.u.	0.001and 0.000
Largest diff. peak and hole	0.486 and −0.417 e/ $Å^3$
$wR2 = \{\Sigma [\omega(F^2 - F^2)^2] / \Sigma [\omega(F^2)^2]\}^{1/2}$	
$R1 = \Sigma \|\|Fo\|-\|Fc\|\| / \Sigma \|Fo\|$	

Table S2. Atomic coordinates and equivalent isotropic displacement parameters for SSB-01-13F1. U(eq) is defined as one third of the trace of the orthogonalized U_{ij} tensor.

Atom	x	y	z	U(eq)
O(1A)	0.3475(6)	0.1932(5)	0.2904(3)	0.0277(14)
O(2A)	0.7344(7)	0.2953(5)	0.5790(4)	0.0274(14)
O(3A)	0.9634(7)	0.4494(5)	0.7100(4)	0.0310(14)
N(1A)	0.8480(9)	0.9118(7)	0.3329(5)	0.0326(18)
N(2A)	0.6538(8)	0.9733(6)	0.4219(5)	0.0277(17)
N(3A)	0.6687(8)	0.9158(6)	0.5513(5)	0.0299(18)
N(4A)	0.6357(9)	1.0305(7)	0.2885(5)	0.041(2)
N(5A)	0.3547(8)	0.3316(6)	0.2008(4)	0.0225(16)
N(6A)	0.4073(7)	0.4541(6)	0.1928(5)	0.0250(16)
C(1A)	0.8679(10)	0.8471(7)	0.4687(6)	0.025(2)
C(2A)	0.9191(10)	0.8512(8)	0.3947(6)	0.029(2)
C(3A)	0.7137(10)	0.9694(8)	0.3499(6)	0.030(2)
C(4A)	0.7304(10)	0.9110(7)	0.4810(6)	0.0234(19)
C(5A)	0.9677(10)	0.7870(7)	0.5397(5)	0.025(2)
C(6A)	0.8915(9)	0.6604(7)	0.5504(6)	0.023(2)
C(7A)	0.7718(10)	0.5799(7)	0.4855(5)	0.0215(19)
C(8A)	0.7139(9)	0.4570(7)	0.4943(5)	0.0196(19)
C(9A)	0.7798(10)	0.4187(7)	0.5716(5)	0.0226(19)
C(10A)	0.9021(10)	0.5003(7)	0.6380(5)	0.025(2)
C(11A)	0.9521(10)	0.6195(8)	0.6272(6)	0.026(2)
C(12A)	0.5902(10)	0.3669(8)	0.4230(5)	0.024(2)
C(13A)	0.5129(10)	0.3948(8)	0.3491(6)	0.025(2)
C(14A)	0.3985(10)	0.3000(8)	0.2802(5)	0.0228(19)
C(15A)	0.2258(9)	0.2443(7)	0.1278(5)	0.025(2)
C(16A)	0.2286(10)	0.1671(8)	−0.0270(5)	0.039(2)
C(17A)	0.2624(11)	0.1918(9)	−0.1045(6)	0.050(2)
C(18A)	0.3317(12)	0.3078(11)	−0.1115(6)	0.053(3)
C(19A)	0.3751(11)	0.4037(9)	−0.0398(6)	0.042(2)
C(20A)	0.3980(9)	0.4733(7)	0.1154(5)	0.0288(18)
C(21A)	0.2686(9)	0.2607(7)	0.0451(5)	0.0299(18)
C(22A)	0.3440(9)	0.3802(7)	0.0383(5)	0.0288(18)
C(23A)	0.0439(9)	0.2687(7)	0.1342(5)	0.0316(19)
C(24A)	−0.1051(10)	0.1822(7)	0.0663(6)	0.038(2)
C(25A)	−0.2818(10)	0.2049(9)	0.0792(7)	0.051(3)
C(26A)	0.6416(12)	0.2743(9)	0.6419(6)	0.039(2)
C(27A)	1.0884(11)	0.5328(8)	0.7783(6)	0.044(2)
O(1B)	0.6479(6)	0.8032(5)	0.7078(4)	0.0294(14)
O(2B)	0.2751(7)	0.7033(5)	0.4216(4)	0.0280(14)
O(3B)	0.0320(7)	0.5543(5)	0.2919(4)	0.0354(15)
N(1B)	0.1477(8)	0.0818(6)	0.6615(5)	0.0291(18)
N(2B)	0.3421(8)	0.0233(6)	0.5734(4)	0.0223(16)
N(3B)	0.3581(9)	−0.0393(6)	0.7031(5)	0.0332(18)
N(4B)	0.3266(8)	0.0866(6)	0.4432(5)	0.0297(18)

Table S2. *Cont.*

Atom	x	y	z	U(eq)
N(5B)	0.6741(8)	0.6639(6)	0.7964(5)	0.0272(17)
N(6B)	0.6453(8)	0.5396(6)	0.8014(5)	0.0299(17)
C(1B)	0.1205(9)	0.1507(7)	0.5240(5)	0.0224(19)
C(2B)	0.0720(10)	0.1417(7)	0.5999(6)	0.025(2)
C(3B)	0.2793(10)	0.0256(7)	0.6435(6)	0.026(2)
C(4B)	0.2660(9)	0.0885(7)	0.5138(5)	0.0222(19)
C(5B)	0.0236(10)	0.2122(7)	0.4565(6)	0.027(2)
C(6B)	0.1026(9)	0.3414(7)	0.4481(5)	0.0216(19)
C(7B)	0.2264(10)	0.4209(7)	0.5133(5)	0.027(2)
C(8B)	0.2892(10)	0.5404(8)	0.5046(5)	0.025(2)
C(9B)	0.2247(10)	0.5825(7)	0.4288(6)	0.025(2)
C(10B)	0.0943(10)	0.5054(8)	0.3627(5)	0.024(2)
C(11B)	0.0376(10)	0.3863(8)	0.3727(5)	0.0239(19)
C(12B)	0.4160(10)	0.6253(8)	0.5739(6)	0.027(2)
C(13B)	0.4902(10)	0.5995(8)	0.6503(5)	0.025(2)
C(14B)	0.6087(10)	0.6944(8)	0.7180(6)	0.025(2)
C(15B)	0.7526(11)	0.7652(8)	0.8705(5)	0.030(2)
C(16B)	0.9543(10)	0.7934(7)	1.0193(5)	0.035(2)
C(17B)	1.0379(9)	0.7456(7)	1.0897(5)	0.033(2)
C(18B)	1.0243(10)	0.6190(8)	1.0875(5)	0.036(2)
C(19B)	0.9227(10)	0.5375(8)	1.0146(5)	0.035(2)
C(20B)	0.7219(10)	0.5042(7)	0.8690(5)	0.0312(19)
C(21B)	0.8524(9)	0.7135(7)	0.9460(5)	0.0299(18)
C(22B)	0.8382(10)	0.5858(7)	0.9452(5)	0.0281(18)
C(23B)	0.6163(10)	0.8387(7)	0.8968(6)	0.038(2)
C(24B)	0.4777(11)	0.7602(9)	0.9237(7)	0.052(3)
C(25B)	0.3387(14)	0.8344(13)	0.9472(8)	0.084(4)
C(26B)	0.3580(11)	0.7180(8)	0.3516(6)	0.033(2)
C(27B)	−0.1522(9)	0.5229(8)	0.2525(5)	0.0322(19)
O(1S)	1.0294(8)	1.0065(6)	0.2169(4)	0.056(2)
O(2S)	−0.0027(10)	−0.0572(8)	0.7678(5)	0.089(3)
O(3S)	0.712(4)	0.158(2)	0.8367(14)	0.155(9)

Table S3. Bond lengths [Å] and angles [°] for SSB-01-13F1.

Bond	Bond Length	Bond	Bond Length
O(2A)-C(9A)	1.391(9)	C(16A)-C(17A)	1.398(12)
O(2A)-C(26A)	1.415(10)	C(16A-H(16A)	0.9500
O(3A)-C(10A)	1.384(10)	C(17A)-C(18A)	1.361(13)
O(3A)-C(27A)	1.437(10)	C(17A-H(17A)	0.9500
N(1A)-C(2A)	1.340(11)	C(18A)-C(19A)	1.393(13)
N(1A)-C(3A)	1.356(10)	C(18A-H(18A)	0.9500
N(2A)-C(4A)	1.336(11)	C(19A)-C(22A)	1.395(11)
N(2A)-C(3A)	1.349(10)	C(19A-H(19A)	0.9500
N(3A)-C(4A)	1.330(10)	C(20A)-C(22A)	1.437(10)

Table S3. *Cont.*

**Bond**	**Bond Length**	**Bond**	**Bond Length**
N(3A-H(3A1)	0.8800	C(20A-H(20A)	0.9500
N(3A-H(3A2)	0.8800	C(21A)-C(22A)	1.412(10)
N(4A)-C(3A)	1.357(10)	C(23A)-C(24A)	1.546(10)
N(4A-H(4A1)	0.8800	C(23A-H(23A)	0.9900
N(4A-H(4A2)	0.8800	C(23A-H(23B)	0.9900
N(5A)-C(14A)	1.362(10)	C(24A)-C(25A)	1.508(11)
N(5A)-N(6A)	1.397(8)	C(24A-H(24A)	0.9900
N(5A)-C(15A)	1.510(10)	C(24A-H(24B)	0.9900
N(6A)-C(20A)	1.289(10)	C(25A-H(25A)	0.9800
C(1A)-C(2A)	1.352(12)	C(25A-H(25B)	0.9800
C(1A)-C(4A)	1.395(11)	C(25A-H(25C)	0.9801
C(1A)-C(5A)	1.526(11)	C(26A-H(26A)	0.9801
C(2A-H(2A)	0.9500	C(26A-H(26B)	0.9800
C(5A)-C(6A)	1.505(11)	C(26A-H(26C)	0.9800
C(5A-H(5A1)	0.9900	C(27A-H(27A)	0.9800
C(5A-H(5A2)	0.9900	C(27A-H(27B)	0.9799
C(6A)-C(7A)	1.376(11)	C(27A-H(27C)	0.9800
C(6A)-C(11A)	1.390(11)	O(1B)-C(14B)	1.245(10)
C(7A)-C(8A)	1.414(11)	O(2B)-C(9B)	1.372(9)
C(7A-H(7A)	0.9500	O(2B)-C(26B)	1.450(10)
C(8A)-C(9A)	1.392(11)	O(3B)-C(10B)	1.357(10)
C(8A)-C(12A)	1.492(11)	O(3B)-C(27B)	1.448(9)
C(9A)-C(10A)	1.402(11)	N(1B)-C(3B)	1.334(9)
C(10A)-C(11A)	1.373(11)	N(1B)-C(2B)	1.344(10)
C(11A-H(11A)	0.9500	N(2B)-C(3B)	1.334(10)
C(12A)-C(13A)	1.317(11)	N(2B)-C(4B)	1.362(10)
C(12A-H(12A)	0.9500	N(3B)-C(3B)	1.367(10)
C(13A)-C(14A)	1.468(11)	N(3B-H(3B1)	0.8800
C(13A-H(13A)	0.9500	N(3B-H(3B2)	0.8800
C(15A)-C(21A)	1.482(11)	N(4B)-C(4B)	1.332(10)
C(15A)-C(23A)	1.524(9)	N(4B-H(4B1)	0.8800
C(15A-H(15A)	1.0000	N(4B-H(4B2)	0.8800
N(5B)-C(14B)	1.368(10)	C(17B)-C(18B)	1.374(11)
N(5B)-N(6B)	1.375(9)	C(17B-H(17B)	0.9500
N(5B)-C(15B)	1.475(11)	C(18B)-C(19B)	1.397(11)
N(6B)-C(20B)	1.268(10)	C(18B-H(18B)	0.9500
C(1B)-C(2B)	1.379(12)	C(19B)-C(22B)	1.386(10)
C(1B)-C(4B)	1.432(10)	C(19B-H(19B)	0.9500
C(1B)-C(5B)	1.487(11)	C(20B)-C(22B)	1.471(11)
C(2B-H(2B)	0.9500	C(20B-H(20B)	0.9500
C(5B)-C(6B)	1.525(11)	C(21B)-C(22B)	1.390(10)
C(5B-H(5B1)	0.9900	C(23B)-C(24B)	1.508(12)
C(5B-H(5B2)	0.9900	C(23B-H(23C)	0.9900
C(6B)-C(7B)	1.388(11)	C(23B-H(23D)	0.9900
C(6B)-C(11B)	1.400(11)	C(24B)-C(25B)	1.520(13)
C(7B)-C(8B)	1.390(11)	C(24B-H(24C)	0.9900
C(7B-H(7B)	0.9500	C(24B-H(24D)	0.9900

Table S3. *Cont.*

Bond	Bond Length	Bond	Bond Length
C(8B)-C(9B)	1.389(12)	C(25B-H(25D)	0.9800
C(8B)-C(12B)	1.459(11)	C(25B-H(25E)	0.9800
C(9B)-C(10B)	1.411(11)	C(25B-H(25F)	0.9799
C(10B)-C(11B)	1.380(11)	C(26B-H(26D)	0.9800
C(11B-H(11B)	0.9500	C(26B-H(26E)	0.9800
C(12B)-C(13B)	1.334(11)	C(26B-H(26F)	0.9800
C(12B-H(12B)	0.9500	C(27B-H(27D)	0.9800
C(13B)-C(14B)	1.471(11)	C(27B-H(27E)	0.9801
C(13B-H(13B)	0.9500	C(27B-H(27F)	0.9799
C(15B)-C(23B)	1.515(11)	O(1S-H(1S1)	0.9570
C(15B)-C(21B)	1.526(11)	O(1S-H(1S2)	0.8840
C(15B-H(15B)	1.0000	O(2S-H(2S1)	0.8700
C(16B)-C(21B)	1.393(11)	O(2S-H(2S2)	0.8580
C(16B)-C(17B)	1.394(11)	O(3S-H(3S1)	0.8610
C(16B-H(16B)	0.9500	O(3S-H(3S2)	0.8660
**Atoms**	**Bond Angle**	**Atoms**	**Bond Angle**
C(9A)-O(2A)-C(26A)	117.2(6)	C(2A)-C(1A)-C(4A)	117.0(8)
C(10A)-O(3A)-C(27A)	114.9(7)	C(2A)-C(1A)-C(5A)	121.5(7)
C(2A)-N(1A)-C(3A)	113.8(7)	C(4A)-C(1A)-C(5A)	121.2(8)
C(4A)-N(2A)-C(3A)	116.9(7)	N(1A)-C(2A)-C(1A)	125.0(8)
C(4A)-N(3A-H(3A1)	120.0	N(1A)-C(2A-H(2A)	117.5
C(4A)-N(3A-H(3A2)	120.0	C(1A)-C(2A-H(2A)	117.5
H(3A1)-N(3A-H(3A2)	120.0	N(2A)-C(3A)-N(1A)	126.2(8)
C(3A)-N(4A-H(4A1)	120.0	N(2A)-C(3A)-N(4A)	117.8(8)
C(3A)-N(4A-H(4A2)	120.0	N(1A)-C(3A)-N(4A)	115.9(8)
H(4A1)-N(4A-H(4A2)	120.0	N(3A)-C(4A)-N(2A)	115.8(7)
C(14A)-N(5A)-N(6A)	118.1(6)	N(3A)-C(4A)-C(1A)	123.1(8)
C(14A)-N(5A)-C(15A)	120.5(7)	N(2A)-C(4A)-C(1A)	121.0(8)
N(6A)-N(5A)-C(15A)	120.5(6)	C(6A)-C(5A)-C(1A)	118.3(7)
C(20A)-N(6A)-N(5A)	116.0(6)	C(6A)-C(5A-H(5A1)	107.7
C(1A)-C(5A-H(5A1)	107.7	C(19A)-C(18A-H(18A)	120.0
C(6A)-C(5A-H(5A2)	107.7	C(18A)-C(19A)-C(22A)	119.4(8)
C(1A)-C(5A-H(5A2)	107.7	C(18A)-C(19A-H(19A)	120.3
H(5A1)-C(5A-H(5A2)	107.1	C(22A)-C(19A-H(19A)	120.3
C(7A)-C(6A)-C(11A)	118.2(8)	N(6A)-C(20A)-C(22A)	125.9(7)
C(7A)-C(6A)-C(5A)	122.8(8)	N(6A)-C(20A-H(20A)	117.0
C(11A)-C(6A)-C(5A)	118.8(7)	C(22A)-C(20A-H(20A)	117.0
C(6A)-C(7A)-C(8A)	121.8(8)	C(16A)-C(21A)-C(22A)	118.5(7)
C(6A)-C(7A-H(7A)	119.1	C(16A)-C(21A)-C(15A)	123.3(7)
C(8A)-C(7A-H(7A)	119.1	C(22A)-C(21A)-C(15A)	118.0(7)
C(9A)-C(8A)-C(7A)	118.3(7)	C(19A)-C(22A)-C(21A)	120.6(7)
C(9A)-C(8A)-C(12A)	119.4(7)	C(19A)-C(22A)-C(20A)	121.4(7)
C(7A)-C(8A)-C(12A)	122.3(7)	C(21A)-C(22A)-C(20A)	117.8(7)
O(2A)-C(9A)-C(8A)	118.9(7)	C(15A)-C(23A)-C(24A)	114.7(6)
O(2A)-C(9A)-C(10A)	120.6(7)	C(15A)-C(23A-H(23A)	108.6
C(8A)-C(9A)-C(10A)	120.2(7)	C(24A)-C(23A-H(23A)	108.6
C(11A)-C(10A)-O(3A)	125.8(8)	C(15A)-C(23A-H(23B)	108.6

Table S3. *Cont.*

Atoms	Bond Angle	Atoms	Bond Angle
C(11A)-C(10A)-C(9A)	119.6(8)	C(24A)-C(23A-H(23B)	108.6
O(3A)-C(10A)-C(9A)	114.7(7)	H(23A)-C(23A-H(23B)	107.6
C(10A)-C(11A)-C(6A)	121.9(8)	C(25A)-C(24A)-C(23A)	112.4(7)
C(10A)-C(11A-H(11A)	119.1	C(25A)-C(24A-H(24A)	109.1
C(6A)-C(11A-H(11A)	119.1	C(23A)-C(24A-H(24A)	109.1
C(13A)-C(12A)-C(8A)	124.5(8)	C(25A)-C(24A-H(24B)	109.1
C(13A)-C(12A-H(12A)	117.7	C(23A)-C(24A-H(24B)	109.1
C(8A)-C(12A-H(12A)	117.7	H(24A)-C(24A-H(24B)	107.9
C(12A)-C(13A)-C(14A)	121.6(8)	C(24A)-C(25A-H(25A)	109.3
C(12A)-C(13A-H(13A)	119.2	C(24A)-C(25A-H(25B)	109.2
C(14A)-C(13A-H(13A)	119.2	H(25A)-C(25A-H(25B)	109.5
O(1A)-C(14A)-N(5A)	119.8(8)	C(24A)-C(25A-H(25C)	109.9
O(1A)-C(14A)-C(13A)	123.3(8)	H(25A)-C(25A-H(25C)	109.5
N(5A)-C(14A)-C(13A)	116.8(8)	H(25B)-C(25A-H(25C)	109.5
C(21A)-C(15A)-N(5A)	109.2(6)	O(2A)-C(26A-H(26A)	109.5
C(21A)-C(15A)-C(23A)	114.7(6)	O(2A)-C(26A-H(26B)	109.6
N(5A)-C(15A)-C(23A)	107.9(6)	H(26A)-C(26A-H(26B)	109.5
C(21A)-C(15A-H(15A)	108.3	O(2A)-C(26A-H(26C)	109.4
N(5A)-C(15A-H(15A)	108.3	H(26A)-C(26A-H(26C)	109.5
C(23A)-C(15A-H(15A)	108.3	H(26B)-C(26A-H(26C)	109.5
C(21A)-C(16A)-C(17A)	120.1(8)	O(3A)-C(27A-H(27A)	109.6
C(21A)-C(16A-H(16A)	120.0	O(3A)-C(27A-H(27B)	109.3
C(17A)-C(16A-H(16A)	120.0	H(27A)-C(27A-H(27B)	109.5
C(18A)-C(17A)-C(16A)	121.3(8)	O(3A)-C(27A-H(27C)	109.5
C(18A)-C(17A-H(17A)	119.3	H(27A)-C(27A-H(27C)	109.5
C(16A)-C(17A-H(17A)	119.3	H(27B)-C(27A-H(27C)	109.5
C(17A)-C(18A)-C(19A)	120.0(9)	C(9B)-O(2B)-C(26B)	115.6(6)
C(17A)-C(18A-H(18A)	120.0	C(10B)-O(3B)-C(27B)	117.1(6)
C(3B)-N(1B)-C(2B)	114.2(7)	C(10B)-C(11B)-C(6B)	121.6(8)
C(3B)-N(2B)-C(4B)	116.4(7)	C(10B)-C(11B-H(11B)	119.2
C(3B)-N(3B-H(3B1)	120.0	C(6B)-C(11B-H(11B)	119.2
C(3B)-N(3B-H(3B2)	120.0	C(13B)-C(12B)-C(8B)	126.1(8)
H(3B1)-N(3B-H(3B2)	120.0	C(13B)-C(12B-H(12B)	117.0
C(4B)-N(4B-H(4B1)	120.0	C(8B)-C(12B-H(12B)	117.0
C(4B)-N(4B-H(4B2)	120.0	C(12B)-C(13B)-C(14B)	121.8(8)
H(4B1)-N(4B-H(4B2)	120.0	C(12B)-C(13B-H(13B)	119.1
C(14B)-N(5B)-N(6B)	116.2(7)	C(14B)-C(13B-H(13B)	119.1
C(14B)-N(5B)-C(15B)	118.8(7)	O(1B)-C(14B)-N(5B)	118.3(8)
N(6B)-N(5B)-C(15B)	124.7(7)	O(1B)-C(14B)-C(13B)	122.6(8)
C(20B)-N(6B)-N(5B)	118.5(7)	N(5B)-C(14B)-C(13B)	119.1(8)
C(2B)-C(1B)-C(4B)	113.8(7)	N(5B)-C(15B)-C(23B)	111.1(7)
C(2B)-C(1B)-C(5B)	123.0(7)	N(5B)-C(15B)-C(21B)	110.8(7)
C(4B)-C(1B)-C(5B)	123.0(7)	C(23B)-C(15B)-C(21B)	111.1(7)
N(1B)-C(2B)-C(1B)	126.2(8)	N(5B)-C(15B-H(15B)	107.9
N(1B)-C(2B-H(2B)	116.9	C(23B)-C(15B-H(15B)	107.9
C(1B)-C(2B-H(2B)	116.9	C(21B)-C(15B-H(15B)	107.9
N(1B)-C(3B)-N(2B)	127.7(8)	C(21B)-C(16B)-C(17B)	120.5(8)

Table S3. *Cont.*

Atoms	Bond Angle	Atoms	Bond Angle
N(1B)-C(3B)-N(3B)	116.6(7)	C(21B)-C(16B-H(16B)	119.7
N(2B)-C(3B)-N(3B)	115.7(7)	C(17B)-C(16B-H(16B)	119.7
N(4B)-C(4B)-N(2B)	117.2(7)	C(18B)-C(17B)-C(16B)	120.7(8)
N(4B)-C(4B)-C(1B)	121.1(8)	C(18B)-C(17B-H(17B)	119.7
N(2B)-C(4B)-C(1B)	121.6(7)	C(16B)-C(17B-H(17B)	119.7
C(1B)-C(5B)-C(6B)	118.0(7)	C(17B)-C(18B)-C(19B)	119.6(8)
C(1B)-C(5B-H(5B1)	107.8	C(17B)-C(18B-H(18B)	120.2
C(6B)-C(5B-H(5B1)	107.8	C(19B)-C(18B-H(18B)	120.2
C(1B)-C(5B-H(5B2)	107.8	C(22B)-C(19B)-C(18B)	119.4(8)
C(6B)-C(5B-H(5B2)	107.8	C(22B)-C(19B-H(19B)	120.3
H(5B1)-C(5B-H(5B2)	107.1	C(18B)-C(19B-H(19B)	120.3
C(7B)-C(6B)-C(11B)	117.9(8)	N(6B)-C(20B)-C(22B)	125.6(7)
C(7B)-C(6B)-C(5B)	123.8(8)	N(6B)-C(20B-H(20B)	117.2
C(11B)-C(6B)-C(5B)	118.2(7)	C(22B)-C(20B-H(20B)	117.2
C(6B)-C(7B)-C(8B)	122.1(8)	C(22B)-C(21B)-C(16B)	118.1(7)
C(6B)-C(7B-H(7B)	118.9	C(22B)-C(21B)-C(15B)	120.9(7)
C(8B)-C(7B-H(7B)	118.9	C(16B)-C(21B)-C(15B)	120.9(7)
C(9B)-C(8B)-C(7B)	119.1(8)	C(19B)-C(22B)-C(21B)	121.7(7)
C(9B)-C(8B)-C(12B)	118.7(8)	C(19B)-C(22B)-C(20B)	121.4(7)
C(7B)-C(8B)-C(12B)	122.2(8)	C(21B)-C(22B)-C(20B)	116.9(7)
O(2B)-C(9B)-C(8B)	120.0(8)	C(24B)-C(23B)-C(15B)	113.1(7)
O(2B)-C(9B)-C(10B)	119.7(8)	C(24B)-C(23B-H(23C)	109.0
C(8B)-C(9B)-C(10B)	120.0(8)	C(15B)-C(23B-H(23C)	109.0
O(3B)-C(10B)-C(11B)	123.8(8)	C(24B)-C(23B-H(23D)	109.0
O(3B)-C(10B)-C(9B)	116.9(7)	C(15B)-C(23B-H(23D)	109.0
C(11B)-C(10B)-C(9B)	119.3(8)	H(23C)-C(23B-H(23D)	107.8
C(23B)-C(24B)-C(25B)	112.5(8)	H(26D)-C(26B-H(26E)	109.5
C(23B)-C(24B-H(24C)	109.1	O(2B)-C(26B-H(26F)	109.5
C(25B)-C(24B-H(24C)	109.1	H(26D)-C(26B-H(26F)	109.5
C(23B)-C(24B-H(24D)	109.1	H(26E)-C(26B-H(26F)	109.5
C(25B)-C(24B-H(24D)	109.1	O(3B)-C(27B-H(27D)	109.1
H(24C)-C(24B-H(24D)	107.8	O(3B)-C(27B-H(27E)	109.7
C(24B)-C(25B-H(25D)	110.0	H(27D)-C(27B-H(27E)	109.5
C(24B)-C(25B-H(25E)	108.4	O(3B)-C(27B-H(27F)	109.6
H(25D)-C(25B-H(25E)	109.5	H(27D)-C(27B-H(27F)	109.5
C(24B)-C(25B-H(25F)	110.0	H(27E)-C(27B-H(27F)	109.5
H(25D)-C(25B-H(25F)	109.5	H(1S1)-O(1S-H(1S2)	122.3
H(25E)-C(25B-H(25F)	109.5	H(2S1)-O(2S-H(2S2)	121.7
O(2B)-C(26B-H(26D)	109.4	H(3S1)-O(3S-H(3S2)	106.2
O(2B)-C(26B-H(26E)	109.5		

Table S4. Anisotropic displacement parameters ($Å^2 \times 10^3$) for SSB-01-13F1. The anisotropic displacement factor exponent takes the form: $-2\pi_2$ (h^2 a × $2U_{11}$ + ... + 2 h k a× b× U_{12}).

Atom	U_{11}	U_{22}	U_{33}	U_{23}	U_{13}	U_{12}
O(1A)	24(3)	27(4)	32(4)	9(3)	4(3)	1(3)
O(2A)	27(3)	27(3)	29(4)	5(3)	5(3)	5(3)
O(3A)	23(3)	35(4)	28(4)	10(3)	−10(3)	−2(3)
N(1A)	30(4)	39(5)	35(5)	16(4)	12(4)	13(4)
N(2A)	20(4)	27(4)	37(5)	7(4)	11(3)	1(3)
N(3A)	19(4)	38(5)	34(5)	9(4)	6(3)	7(3)
N(4A)	38(5)	56(5)	41(5)	28(4)	14(4)	22(4)
N(5A)	21(3)	19(4)	26(4)	2(3)	3(3)	2(3)
N(6A)	12(3)	22(4)	38(5)	7(3)	3(3)	−6(3)
C(1A)	21(4)	20(5)	35(5)	7(4)	4(4)	1(4)
C(2A)	25(5)	26(5)	35(6)	3(4)	7(4)	8(4)
C(3A)	23(5)	31(5)	36(6)	12(4)	5(4)	−1(4)
C(4A)	19(4)	18(5)	32(5)	3(4)	6(4)	−6(3)
C(5A)	26(5)	16(5)	34(6)	5(4)	9(4)	−1(4)
C(6A)	13(4)	23(5)	35(6)	7(4)	7(4)	2(4)
C(7A)	20(4)	23(5)	21(5)	5(4)	2(4)	7(4)
C(8A)	14(4)	21(5)	25(5)	6(4)	4(4)	8(3)
C(9A)	20(4)	20(5)	29(5)	7(4)	6(4)	4(4)
C(10A)	19(4)	24(5)	34(6)	9(4)	6(4)	5(4)
C(11A)	18(4)	29(5)	27(5)	−5(4)	5(4)	5(4)
C(12A)	20(4)	29(5)	27(5)	6(4)	12(4)	6(4)
C(13A)	18(4)	23(5)	32(6)	2(4)	5(4)	1(4)
C(14A)	16(4)	30(5)	26(5)	10(4)	8(4)	4(4)
C(15A)	9(4)	26(5)	33(5)	0(4)	−4(4)	−5(3)
C(16A)	31(5)	44(5)	35(5)	−6(4)	4(4)	5(4)
C(17A)	39(5)	62(7)	37(6)	−15(5)	0(4)	10(5)
C(18A)	42(6)	85(8)	32(6)	13(5)	9(4)	7(5)
C(19A)	32(5)	58(6)	38(6)	12(5)	12(4)	8(4)
C(20A)	26(4)	25(4)	39(5)	10(4)	10(4)	2(3)
C(21A)	16(4)	38(5)	34(5)	4(4)	0(3)	11(3)
C(22A)	23(4)	33(5)	32(5)	10(4)	5(4)	7(3)
C(23A)	17(4)	36(4)	36(5)	1(4)	−2(3)	−1(3)
C(24A)	26(4)	30(4)	47(6)	−6(4)	1(4)	1(3)
C(25A)	18(4)	49(6)	78(7)	1(5)	4(4)	−1(4)
C(26A)	32(5)	38(6)	43(6)	3(5)	8(5)	−5(4)
C(27A)	31(5)	56(6)	39(6)	10(5)	−4(4)	−1(4)
O(1B)	23(3)	23(3)	42(4)	10(3)	7(3)	0(3)
O(2B)	28(3)	23(3)	32(4)	10(3)	4(3)	1(3)
O(3B)	22(3)	42(4)	42(4)	24(3)	−1(3)	−5(3)
N(1B)	27(4)	18(4)	45(5)	6(4)	14(4)	4(3)
N(2B)	18(3)	22(4)	27(4)	4(3)	5(3)	−1(3)
N(3B)	31(4)	35(4)	41(5)	17(4)	15(4)	14(3)

Table S4. *Cont.*

Atom	U_{11}	U_{22}	U_{33}	U_{23}	U_{13}	U_{12}
N(4B)	23(4)	35(4)	39(5)	17(4)	12(4)	12(3)
N(5B)	26(4)	23(4)	32(5)	6(3)	6(3)	0(3)
N(6B)	30(4)	26(4)	33(4)	10(3)	6(3)	−2(3)
C(1B)	17(4)	18(5)	31(5)	4(4)	5(4)	−2(3)
C(2B)	21(4)	17(4)	40(6)	8(4)	11(4)	−2(4)
C(3B)	23(5)	17(5)	34(6)	0(4)	7(4)	−2(4)
C(4B)	15(4)	23(5)	30(5)	8(4)	6(4)	0(3)
C(5B)	19(4)	25(5)	37(6)	4(4)	7(4)	6(4)
C(6B)	15(4)	20(4)	27(5)	1(4)	2(4)	1(3)
C(7B)	19(4)	31(5)	31(6)	8(4)	5(4)	7(4)
C(8B)	22(5)	25(5)	28(5)	6(4)	9(4)	3(4)
C(9B)	20(4)	16(5)	40(6)	7(4)	10(4)	5(4)
C(10B)	12(4)	35(5)	28(5)	11(4)	6(4)	7(4)
C(11B)	18(4)	27(5)	26(5)	9(4)	5(4)	−3(4)
C(12B)	18(4)	24(5)	36(6)	2(4)	4(4)	2(4)
C(13B)	23(4)	26(5)	24(5)	2(4)	4(4)	−3(4)
C(14B)	23(5)	24(5)	30(6)	5(4)	6(4)	7(4)
C(15B)	34(5)	26(5)	29(5)	6(4)	6(4)	1(4)
C(16B)	31(4)	31(4)	43(5)	2(4)	13(4)	5(4)
C(17B)	22(4)	39(5)	37(5)	11(4)	1(4)	−1(4)
C(18B)	28(4)	49(6)	35(5)	17(4)	7(4)	12(4)
C(19B)	29(4)	35(5)	42(5)	15(4)	5(4)	1(4)
C(20B)	27(4)	28(4)	41(5)	13(4)	12(4)	−1(3)
C(21B)	22(4)	39(5)	31(5)	7(4)	10(4)	10(4)
C(22B)	25(4)	30(5)	28(5)	8(4)	4(4)	2(3)
C(23B)	34(5)	32(5)	47(6)	4(4)	8(4)	12(4)
C(24B)	36(5)	53(6)	67(7)	4(5)	18(5)	9(4)
C(25B)	48(7)	111(10)	111(11)	26(8)	44(7)	35(7)
C(26B)	27(5)	28(5)	52(7)	20(5)	16(5)	7(4)
C(27B)	15(4)	40(5)	40(5)	12(4)	−2(4)	7(3)
O(1S)	61(4)	58(4)	39(4)	1(3)	16(3)	−40(3)
O(2S)	66(5)	121(6)	64(5)	32(4)	0(4)	−53(4)
O(3S)	200(30)	108(17)	140(20)	30(14)	0(18)	26(17)

Table S5. Hydrogen coordinates and isotropic displacement parameters for SSB-01-13F1.

Hydrogen	x	y	z	U(eq)
H(3A1)	0.5826	0.9581	0.5566	0.036
H(3A2)	0.7140	0.8765	0.5925	0.036
H(4A1)	0.5492	1.0709	0.2964	0.050
H(4A2)	0.6718	1.0297	0.2408	0.050
H(2A)	1.0127	0.8079	0.3858	0.034
H(5A1)	1.0847	0.7805	0.5295	0.030
H(5A2)	0.9836	0.8449	0.5951	0.030
H(7A)	0.7268	0.6075	0.4333	0.026
H(11A)	1.0304	0.6756	0.6736	0.031
H(12A)	0.5655	0.2836	0.4316	0.029
H(13A)	0.5318	0.4784	0.3401	0.030
H(15A)	0.2374	0.1566	0.1358	0.030
H(16A)	0.1781	0.0859	−0.0239	0.046
H(17A)	0.2365	0.1265	−0.1533	0.060
H(18A)	0.3506	0.3233	−0.1651	0.064
H(19A)	0.4254	0.4844	−0.0440	0.050
H(20A)	0.4297	0.5567	0.1088	0.035
H(23A)	0.0332	0.3564	0.1283	0.038
H(23B)	0.0299	0.2591	0.1923	0.038
H(24A)	−0.0971	0.1958	0.0081	0.045
H(24B)	−0.0915	0.0941	0.0696	0.045
H(25A)	−0.2946	0.1833	0.1343	0.076
H(25B)	−0.3715	0.1526	0.0322	0.076
H(25C)	−0.2943	0.2930	0.0793	0.076
H(26A)	0.7097	0.3177	0.6987	0.058
H(26B)	0.6202	0.1846	0.6420	0.058
H(26C)	0.5304	0.3063	0.6284	0.058
H(27A)	1.1938	0.5550	0.7588	0.066
H(27B)	1.1174	0.4915	0.8281	0.066
H(27C)	1.0392	0.6086	0.7947	0.066
H(3B1)	0.3213	−0.0424	0.7503	0.040
H(3B2)	0.4455	−0.0781	0.6942	0.040
H(4B1)	0.4122	0.0444	0.4366	0.036
H(4B2)	0.2810	0.1275	0.4031	0.036
H(2B)	−0.0239	0.1816	0.6096	0.030
H(5B1)	−0.0930	0.2187	0.4672	0.033
H(5B2)	0.0068	0.1562	0.4002	0.033
H(7B)	0.2696	0.3927	0.5655	0.032
H(11B)	−0.0475	0.3335	0.3273	0.029
H(12B)	0.4489	0.7065	0.5636	0.032
H(13B)	0.4658	0.5175	0.6612	0.030
H(15B)	0.8373	0.8234	0.8524	0.036
H(16B)	0.9668	0.8811	1.0212	0.042
H(17B)	1.1049	0.8012	1.1398	0.040

Table S5. *Cont.*

Hydrogen	x	y	z	U(eq)
H(18B)	1.0836	0.5871	1.1353	0.043
H(19B)	0.9117	0.4498	1.0126	0.042
H(20B)	0.7025	0.4176	0.8706	0.037
H(23C)	0.6738	0.9075	0.9452	0.045
H(23D)	0.5608	0.8765	0.8478	0.045
H(24C)	0.5324	0.7247	0.9740	0.062
H(24D)	0.4224	0.6899	0.8761	0.062
H(25D)	0.3903	0.9010	0.9969	0.126
H(25E)	0.2506	0.7775	0.9617	0.126
H(25F)	0.2844	0.8710	0.8982	0.126
H(26D)	0.4831	0.7170	0.3713	0.050
H(26E)	0.3384	0.7977	0.3334	0.050
H(26F)	0.3088	0.6493	0.3030	0.050
H(27D)	−0.1741	0.4400	0.2160	0.048
H(27E)	−0.1900	0.5847	0.2173	0.048
H(27F)	−0.2169	0.5221	0.2975	0.048
H(1S1)	0.9525	0.9815	0.2511	0.068
H(1S2)	1.1240	1.0617	0.2387	0.068
H(2S1)	0.0432	−0.0138	0.7354	0.107
H(2S2)	−0.1058	−0.0978	0.7503	0.107
H(3S1)	0.6106	0.1768	0.8383	0.186
H(3S2)	0.7803	0.2277	0.8455	0.186

Table S6. Torsion angles [°] for SSB-01-13F1.

Atoms	Torsion Angle
C(14A)-N(5A)-N(6A)-C(20A)	165.2(7)
C(15A)-N(5A)-N(6A)-C(20A)	−26.0(9)
C(3A)-N(1A)-C(2A)-C(1A)	−1.0(12)
C(4A)-C(1A)-C(2A)-N(1A)	0.0(13)
C(5A)-C(1A)-C(2A)-N(1A)	−173.8(8)
C(4A)-N(2A)-C(3A)-N(1A)	−2.4(12)
C(4A)-N(2A)-C(3A)-N(4A)	179.0(7)
C(2A)-N(1A)-C(3A)-N(2A)	2.3(12)
C(2A)-N(1A)-C(3A)-N(4A)	−179.1(8)
C(3A)-N(2A)-C(4A)-N(3A)	−179.7(7)
C(3A)-N(2A)-C(4A)-C(1A)	1.1(11)
C(2A)-C(1A)-C(4A)-N(3A)	−179.2(8)
C(5A)-C(1A)-C(4A)-N(3A)	−5.4(12)
C(2A)-C(1A)-C(4A)-N(2A)	0.0(12)
C(5A)-C(1A)-C(4A)-N(2A)	173.7(7)
C(2A)-C(1A)-C(5A)-C(6A)	−104.5(9)
C(4A)-C(1A)-C(5A)-C(6A)	82.0(10)
C(1A)-C(5A)-C(6A)-C(7A)	21.6(11)
C(1A)-C(5A)-C(6A)-C(11A)	−162.5(7)
C(11A)-C(6A)-C(7A)-C(8A)	−1.8(11)

Table S6. *Cont.*

Atoms	Torsion Angle
C(5A)-C(6A)-C(7A)-C(8A)	174.1(7)
C(6A)-C(7A)-C(8A)-C(9A)	0.7(11)
C(6A)-C(7A)-C(8A)-C(12A)	−177.2(7)
C(26A)-O(2A)-C(9A)-C(8A)	−118.0(8)
C(26A)-O(2A)-C(9A)-C(10A)	67.7(9)
C(7A)-C(8A)-C(9A)-O(2A)	−175.1(6)
C(12A)-C(8A)-C(9A)-O(2A)	2.9(10)
C(7A)-C(8A)-C(9A)-C(10A)	−0.8(10)
C(12A)-C(8A)-C(9A)-C(10A)	177.1(6)
C(27A)-O(3A)-C(10A)-C(11A)	0.9(11)
C(27A)-O(3A)-C(10A)-C(9A)	−179.8(7)
O(2A)-C(9A)-C(10A)-C(11A)	176.3(7)
C(8A)-C(9A)-C(10A)-C(11A)	2.1(11)
O(2A)-C(9A)-C(10A)-O(3A)	−3.1(10)
C(8A)-C(9A)-C(10A)-O(3A)	−177.2(7)
O(3A)-C(10A)-C(11A)-C(6A)	176.0(7)
C(9A)-C(10A)-C(11A)-C(6A)	−3.3(11)
C(7A)-C(6A)-C(11A)-C(10A)	3.1(11)
C(5A)-C(6A)-C(11A)-C(10A)	−173.0(7)
C(9A)-C(8A)-C(12A)-C(13A)	176.3(7)
C(7A)-C(8A)-C(12A)-C(13A)	−5.9(11)
C(8A)-C(12A)-C(13A)-C(14A)	177.3(7)
N(6A)-N(5A)-C(14A)-O(1A)	176.9(6)
C(15A)-N(5A)-C(14A)-O(1A)	8.1(10)
N(6A)-N(5A)-C(14A)-C(13A)	−6.0(9)
C(15A)-N(5A)-C(14A)-C(13A)	−174.9(6)
C(12A)-C(13A)-C(14A)-O(1A)	10.3(11)
C(12A)-C(13A)-C(14A)-N(5A)	−166.6(7)
C(14A)-N(5A)-C(15A)-C(21A)	−149.6(7)
N(6A)-N(5A)-C(15A)-C(21A)	41.8(8)
C(14A)-N(5A)-C(15A)-C(23A)	85.1(8)
N(6A)-N(5A)-C(15A)-C(23A)	−83.5(8)
C(21A)-C(16A)-C(17A)-C(18A)	−1.2(13)
C(16A)-C(17A)-C(18A)-C(19A)	1.9(14)
C(17A)-C(18A)-C(19A)-C(22A)	−1.1(13)
N(5A)-N(6A)-C(20A)-C(22A)	−2.0(10)
C(17A)-C(16A)-C(21A)-C(22A)	−0.2(11)
C(17A)-C(16A)-C(21A)-C(15A)	175.7(7)
N(5A)-C(15A)-C(21A)-C(16A)	152.6(7)
C(23A)-C(15A)-C(21A)-C(16A)	−86.2(9)
N(5A)-C(15A)-C(21A)-C(22A)	−31.6(9)
C(23A)-C(15A)-C(21A)-C(22A)	89.6(8)
C(18A)-C(19A)-C(22A)-C(21A)	−0.2(12)
C(18A)-C(19A)-C(22A)-C(20A)	175.4(8)

Table S6. *Cont.*

Atoms	Torsion Angle
C(16A)-C(21A)-C(22A)-C(19A)	0.9(11)
C(15A)-C(21A)-C(22A)-C(19A)	−175.2(7)
C(16A)-C(21A)-C(22A)-C(20A)	−174.9(7)
C(15A)-C(21A)-C(22A)-C(20A)	9.0(10)
N(6A)-C(20A)-C(22A)-C(19A)	−165.2(7)
N(6A)-C(20A)-C(22A)-C(21A)	10.5(11)
C(21A)-C(15A)-C(23A)-C(24A)	60.9(9)
N(5A)-C(15A)-C(23A)-C(24A)	−177.2(7)
C(15A)-C(23A)-C(24A)-C(25A)	176.3(8)
C(14B)-N(5B)-N(6B)-C(20B)	171.0(7)
C(15B)-N(5B)-N(6B)-C(20B)	−15.6(11)
C(3B)-N(1B)-C(2B)-C(1B)	−1.3(11)
C(4B)-C(1B)-C(2B)-N(1B)	0.0(11)
C(5B)-C(1B)-C(2B)-N(1B)	176.2(7)
C(2B)-N(1B)-C(3B)-N(2B)	0.4(11)
C(2B)-N(1B)-C(3B)-N(3B)	−178.4(7)
C(4B)-N(2B)-C(3B)-N(1B)	1.7(12)
C(4B)-N(2B)-C(3B)-N(3B)	−179.4(7)
C(3B)-N(2B)-C(4B)-N(4B)	−179.6(7)
C(3B)-N(2B)-C(4B)-C(1B)	−3.1(11)
C(2B)-C(1B)-C(4B)-N(4B)	178.7(7)
C(5B)-C(1B)-C(4B)-N(4B)	2.5(11)
C(2B)-C(1B)-C(4B)-N(2B)	2.4(11)
C(5B)-C(1B)-C(4B)-N(2B)	−173.9(7)
C(2B)-C(1B)-C(5B)-C(6B)	104.7(9)
C(4B)-C(1B)-C(5B)-C(6B)	−79.4(10)
C(1B)-C(5B)-C(6B)-C(7B)	−20.7(11)
C(1B)-C(5B)-C(6B)-C(11B)	164.1(7)
C(11B)-C(6B)-C(7B)-C(8B)	−1.6(11)
C(5B)-C(6B)-C(7B)-C(8B)	−176.8(7)
C(6B)-C(7B)-C(8B)-C(9B)	0.8(11)
C(6B)-C(7B)-C(8B)-C(12B)	178.4(7)
C(26B)-O(2B)-C(9B)-C(8B)	121.6(8)
C(26B)-O(2B)-C(9B)-C(10B)	−64.8(9)
C(7B)-C(8B)-C(9B)-O(2B)	175.0(7)
C(12B)-C(8B)-C(9B)-O(2B)	−2.7(11)
C(7B)-C(8B)-C(9B)-C(10B)	1.5(11)
C(12B)-C(8B)-C(9B)-C(10B)	−176.2(7)
C(27B)-O(3B)-C(10B)-C(11B)	38.0(11)
C(27B)-O(3B)-C(10B)-C(9B)	−142.2(7)
O(2B)-C(9B)-C(10B)-O(3B)	3.7(10)
C(8B)-C(9B)-C(10B)-O(3B)	177.3(7)
O(2B)-C(9B)-C(10B)-C(11B)	−176.4(6)
C(8B)-C(9B)-C(10B)-C(11B)	−2.8(11)
O(3B)-C(10B)-C(11B)-C(6B)	−178.2(7)

Table S6. *Cont.*

Atoms	Torsion Angle
C(9B)-C(10B)-C(11B)-C(6B)	2.0(11)
C(7B)-C(6B)-C(11B)-C(10B)	0.2(11)
C(5B)-C(6B)-C(11B)-C(10B)	175.7(7)
C(9B)-C(8B)-C(12B)-C(13B)	−180.0(8)
C(7B)-C(8B)-C(12B)-C(13B)	2.4(12)
C(8B)-C(12B)-C(13B)-C(14B)	−176.1(7)
N(6B)-N(5B)-C(14B)-O(1B)	−172.1(7)
C(15B)-N(5B)-C(14B)-O(1B)	14.0(10)
N(6B)-N(5B)-C(14B)-C(13B)	10.1(10)
C(15B)-N(5B)-C(14B)-C(13B)	−163.7(7)
C(12B)-C(13B)-C(14B)-O(1B)	−0.8(12)
C(12B)-C(13B)-C(14B)-N(5B)	176.9(7)
C(14B)-N(5B)-C(15B)-C(23B)	68.5(9)
N(6B)-N(5B)-C(15B)-C(23B)	−104.7(8)
C(14B)-N(5B)-C(15B)-C(21B)	−167.5(6)
N(6B)-N(5B)-C(15B)-C(21B)	19.3(10)
C(21B)-C(16B)-C(17B)-C(18B)	1.3(11)
C(16B)-C(17B)-C(18B)-C(19B)	−1.3(11)
C(17B)-C(18B)-C(19B)-C(22B)	0.5(11)
N(5B)-N(6B)-C(20B)-C(22B)	1.5(11)
C(17B)-C(16B)-C(21B)-C(22B)	−0.5(10)
C(17B)-C(16B)-C(21B)-C(15B)	176.7(7)
N(5B)-C(15B)-C(21B)-C(22B)	−10.5(10)
C(23B)-C(15B)-C(21B)-C(22B)	113.5(8)
N(5B)-C(15B)-C(21B)-C(16B)	172.4(7)
C(23B)-C(15B)-C(21B)-C(16B)	−63.6(9)
C(18B)-C(19B)-C(22B)-C(21B)	0.3(11)
C(18B)-C(19B)-C(22B)-C(20B)	−176.5(7)
C(16B)-C(21B)-C(22B)-C(19B)	−0.3(11)
C(15B)-C(21B)-C(22B)-C(19B)	−177.5(7)
C(16B)-C(21B)-C(22B)-C(20B)	176.6(7)
C(15B)-C(21B)-C(22B)-C(20B)	−0.6(10)
N(6B)-C(20B)-C(22B)-C(19B)	−176.9(8)
N(6B)-C(20B)-C(22B)-C(21B)	6.2(11)
N(5B)-C(15B)-C(23B)-C(24B)	60.6(10)
C(21B)-C(15B)-C(23B)-C(24B)	−63.2(9)
C(15B)-C(23B)-C(24B)-C(25B)	−178.2(9)

Table S7. Hydrogen bonds for SSB-01-13F1[Å and °].

D-H...A	d(D-H)	d(H...A)	d(D...A)	<(DHA)
N(3A-H(3A1)...N(2B)#1	0.88	2.17	3.036(9)	167.3
N(3A-H(3A2)...O(1B)	0.88	2.28	3.009(9)	140.1
N(4A-H(4A1)...O(1A)#1	0.88	2.19	3.055(9)	165.9
C(2A-H(2A)...O(2B)#2	0.95	2.49	3.393(10)	158.7
C(26A-H(26A)...O(3A)	0.98	2.31	2.924(11)	119.7
C(26A-H(26B)...N(2B)	0.98	2.60	3.318(11)	130.1
N(3B-H(3B1)...O(2S)	0.88	2.65	3.265(10)	127.9
N(3B-H(3B2)...O(1B)#3	0.88	2.17	3.021(8)	161.8
N(4B-H(4B1)...N(2A)#3	0.88	2.20	3.067(9)	169.3
N(4B-H(4B2)...O(1A)	0.88	2.21	2.919(9)	137.9
C(2B-H(2B)...O(2A)#4	0.95	2.41	3.305(10)	157.8
C(15B-H(15B)...O(2S)#5	1.00	2.48	3.458(11)	164.3
C(23B-H(23D)...O(1B)	0.99	2.55	3.092(10)	114.2
C(26B-H(26F)...O(3B)	0.98	2.30	2.879(10)	117.3
O(1S-H(1S1)...N(1A)	0.96	1.92	2.856(9)	165.2
O(1S-H(1S2)...O(1A)#5	0.88	2.09	2.978(8)	179.8
O(2S-H(2S1)...N(1B)	0.87	1.96	2.834(10)	179.1
O(2S-H(2S2)...O(1B)#6	0.86	2.06	2.916(9)	179.4

Symmetry transformations used to generate equivalent atoms: #1 x, y + 1, z #2 x + 1, y, z #3 x, y − 1, z #4 x − 1, y, z, #5 x + 1, y + 1, z #6 x − 1, y − 1, z.
